# A comparative study of HSO-optimized PID and TID controllers with fuzzy adaptation for delta robot control

**DOI:** 10.1038/s41598-026-63558-0

**Published:** 2026-08-16

**Authors:** Donia Saleem, Hasan Eleashy, Mohamed A. Shamseldin

**Affiliations:** Department of Mechanical Engineering, Future University in Egypt, Cairo, Egypt

**Keywords:** Delta robot, PID control, Tilt-Integral-Derivative control, Fuzzy self-tuning, Harmony Search Optimization, Simscape, System identification, NLARX, Trajectory tracking, Engineering, Mathematics and computing

## Abstract

This paper presents the development, tuning, and comparative evaluation of intelligent control strategies for a highly nonlinear Delta robot. The work includes the development and validation of a Simscape-based model derived from the experimental platform, followed by controller design using four approaches: Harmony-Search-optimized PID, Harmony-Search-optimized Tilt-Integral-Derivative (TID), self-tuning fuzzy PID, and self-tuning fuzzy TID control. System identification was used to obtain a reduced model suitable for efficient controller tuning, and the identified NLARX model achieved an MSE of 0.02662. Controller performance was evaluated using step, sinusoidal, and repeating-sequence stair inputs. The step-response results show that optimized TID substantially reduces steady-state error compared with optimized PID (0.2982 versus 2.2705 for θ₁), although with higher overshoot in some cases. For sinusoidal trajectory tracking, the fuzzy TID controller achieved the lowest or nearly lowest RMSE values in the Cartesian directions, including 0.2602 mm, 0.3046 mm, and 0.6548 mm in X, Y, and Z, respectively. For repeating-sequence stair inputs, fuzzy TID again achieved the lowest RMSE across the three axes. Overall, fuzzy TID provides the best accuracy and smoothness, while optimized TID remains a strong lower-complexity alternative for real-time implementation.

## Introduction

Robotics has become a cornerstone of modern engineering, integrating principles from mechanics, control theory, computer science, and artificial intelligence to design intelligent systems capable of performing complex and precise tasks. The theoretical foundations of robotics, including kinematics, dynamics, and motion planning, are well established in the literature and form the basis for the development of advanced robotic systems^1–4^. These principles enable robots to operate efficiently in structured and unstructured environments, supporting a wide range of applications across multiple domains.

Over the past few decades, robotics has witnessed rapid expansion into various sectors, including industrial automation, military operations, service applications, and agriculture. Industrial robots are widely used in manufacturing due to their accuracy, repeatability, and productivity^5^, while mobile robots have been extensively studied for navigation, exploration, and logistics tasks^6^. In military contexts, robots are deployed for hazardous missions, reducing risks to human operators^7,8^. Service robots have also gained significant attention for their roles in healthcare, domestic assistance, and human–robot interaction^9–11^. Furthermore, the integration of robotics into agriculture has led to the emergence of smart farming technologies, improving efficiency and sustainability through automation^12,13^–^14,15^. Commercial robotic solutions, including multi-axis articulated robots, have also become increasingly accessible, further accelerating industrial adoption^16^.

Among different robotic architectures, manipulators are broadly classified into serial and parallel configurations. Parallel manipulators have attracted considerable interest due to their high stiffness, better load-carrying capacity, and superior dynamic performance compared to their serial counterparts^4^. The Delta robot, a well-known parallel manipulator, has gained widespread popularity for high-speed and high-precision applications such as pick-and-place operations, packaging, and additive manufacturing^17–20^. Its lightweight structure and closed-loop kinematic chains allow for rapid motion and improved accuracy. Extensive studies have been conducted on the kinematic and dynamic modeling of Delta robots, including forward and inverse kinematics, Jacobian analysis, and workspace determination^21–27^. Additionally, design optimization, stiffness analysis, and dynamic performance evaluation have been explored to enhance the capabilities of Delta robots^28,29^–^30^.

The control of robotic manipulators remains a critical challenge due to nonlinear dynamics, parameter uncertainties, and external disturbances. Classical control techniques such as Proportional–Integral–Derivative (PID) controllers are widely used due to their simplicity and effectiveness in industrial applications^31,32^. However, advanced control strategies have been developed to improve performance, including computed torque control, adaptive control, sliding mode control, and impedance control^33–38^. Intelligent control approaches based on artificial intelligence, such as neural networks, fuzzy logic, and support vector machines, have also demonstrated significant potential in enhancing tracking performance and robustness^39^^40,41^,–^42,43^. Hybrid and optimization-based control methods, including fuzzy LQR, ant colony optimization, and particle swarm optimization, have been proposed to further improve system stability and efficiency^44–46^.

Recent advancements have focused on integrating machine learning and optimization techniques into robotic control systems. Neural network-based controllers and real-time learning approaches have shown promising results in addressing uncertainties and improving trajectory tracking accuracy^41,47,48^. Moreover, trajectory planning and optimization methods, such as time-optimal control and heuristic algorithms, have been applied to enhance the efficiency of robotic motion^44,49–51^. System identification techniques also play a vital role in modeling and controller design, enabling accurate representation of dynamic systems^52,53^. These developments have contributed to the emergence of intelligent and adaptive robotic systems capable of operating in complex environments.

In addition to control strategies, advancements in sensing, perception, and software frameworks have further enhanced robotic capabilities. Vision-based control, gesture recognition, and integration with robotic operating systems (ROS) have enabled more intuitive and flexible human–robot interaction^54–56^. Experimental and simulation studies have also been conducted to validate model-based control approaches and improve system performance^57^. Furthermore, modern robotic systems increasingly rely on advanced controller tuning methods and servo identification techniques to achieve high precision and stability^58^.

Despite these advancements, several challenges remain in achieving robust, high-speed, and accurate control of parallel manipulators, particularly Delta robots. Issues such as nonlinear coupling, parameter uncertainties, and external disturbances continue to limit performance. To address these challenges, various adaptive, robust, and intelligent control techniques have been proposed, including feedforward compensation, disturbance rejection, and iterative learning control^59–62^. Additionally, recent research has explored novel control paradigms such as fractional-order controllers, tilt-integral-derivative controllers, and adaptive compensation schemes to further enhance system performance^63–65^–^66^.

Moreover, modern robotic applications extend beyond traditional industrial tasks to include advanced systems such as multi-degree-of-freedom manipulators, bio-inspired robots, and assistive technologies^67–71^. These applications require sophisticated modeling, optimization, and control techniques to ensure safe and efficient operation. Optimization methods, including numerical optimization and heuristic algorithms, are widely used to design and tune robotic systems for improved performance^50,51,72^.

Predictive control provides an important advanced benchmark. Predictive functional control and dynamic matrix control can explicitly use a process model, future predictions, constraints, and multi-objective criteria. Recent auxiliary-variable predictive functional control and co-optimized dynamic matrix control formulations improve set-point tracking and performance optimization^73,74^. Such methods are attractive when an accurate online model and sufficient computational resources are available. The present work does not claim that PID/TID is universally superior to model-predictive control. Instead, PID/TID structures are studied because they are transparent, compatible with standard servo-drive architectures, and inexpensive to execute at high sampling rates; the identified model is used for offline tuning rather than repeated online optimization.

In this context, this paper presents a comprehensive study of the modelling, identification, optimization, and trajectory control of a Delta parallel robot. The study evaluates tracking accuracy, transient behaviour, and computationally practical controller structures. External-disturbance robustness is not inferred from reference-tracking tests alone and is treated separately in the limitations. The remainder of the paper is organized as follows: Sect. 2 presents the modelling and optimization methodology, Sect. 3 describes the controllers, Sect. 4 discusses the comparative results, and the final sections summarize the findings and limitations.

## Methodology and control techniques

This section details the modeling and control methodology. A Simscape model was developed from the experimental hardware and SolidWorks representation of the Delta robot. System identification was then used to validate and simplify the dynamic representation so that optimization could be performed efficiently. Four controllers were implemented and compared: optimized PID, optimized TID, self-tuning fuzzy PID, and self-tuning fuzzy TID. The optimized controllers were tuned using Harmony Search Optimization, and their responses were evaluated under multiple reference inputs.

### Model initiation

**Fig. 1:**
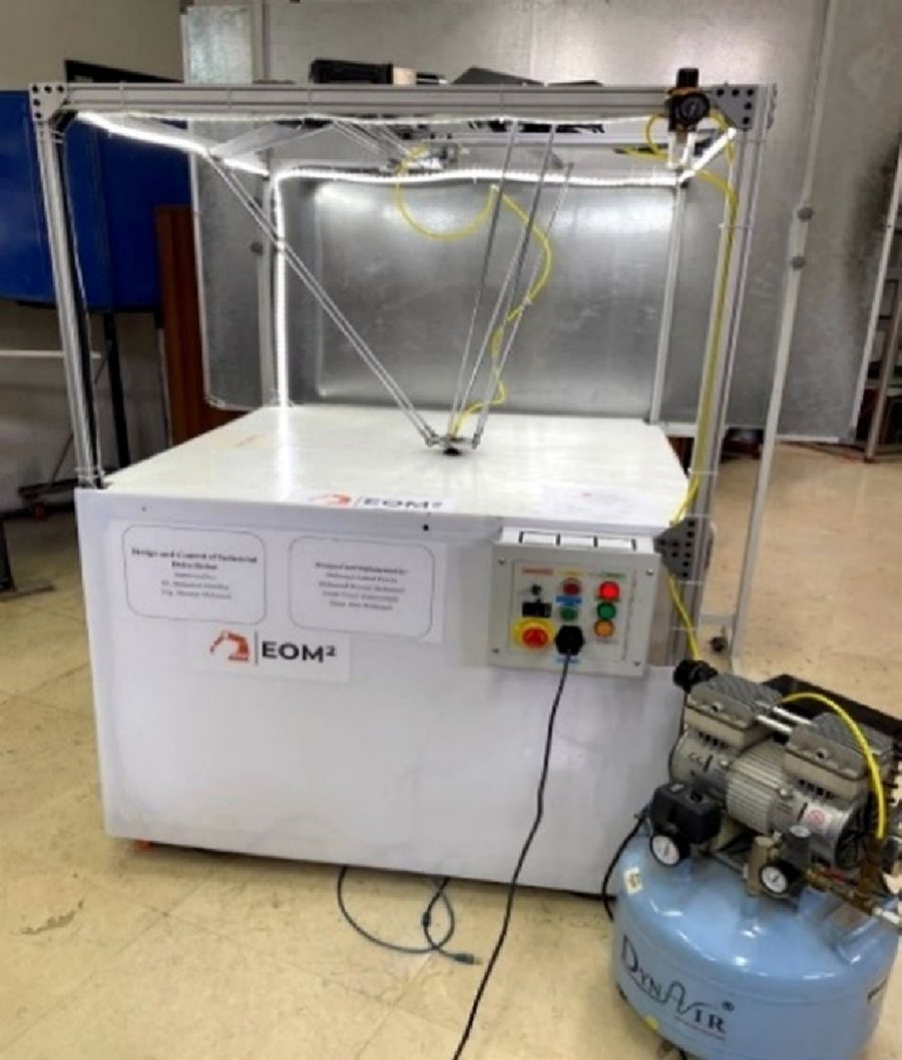
Actual model.

To enhance the performance of the Delta robot, multiple control techniques are implemented and compared to determine the most effective strategy based on system response. An existing Delta robot system located in the laboratories of Future University in Egypt (FUE), as shown in Fig. 1, is utilized as the experimental platform for applying and evaluating these control approaches.

**Fig. 2:**
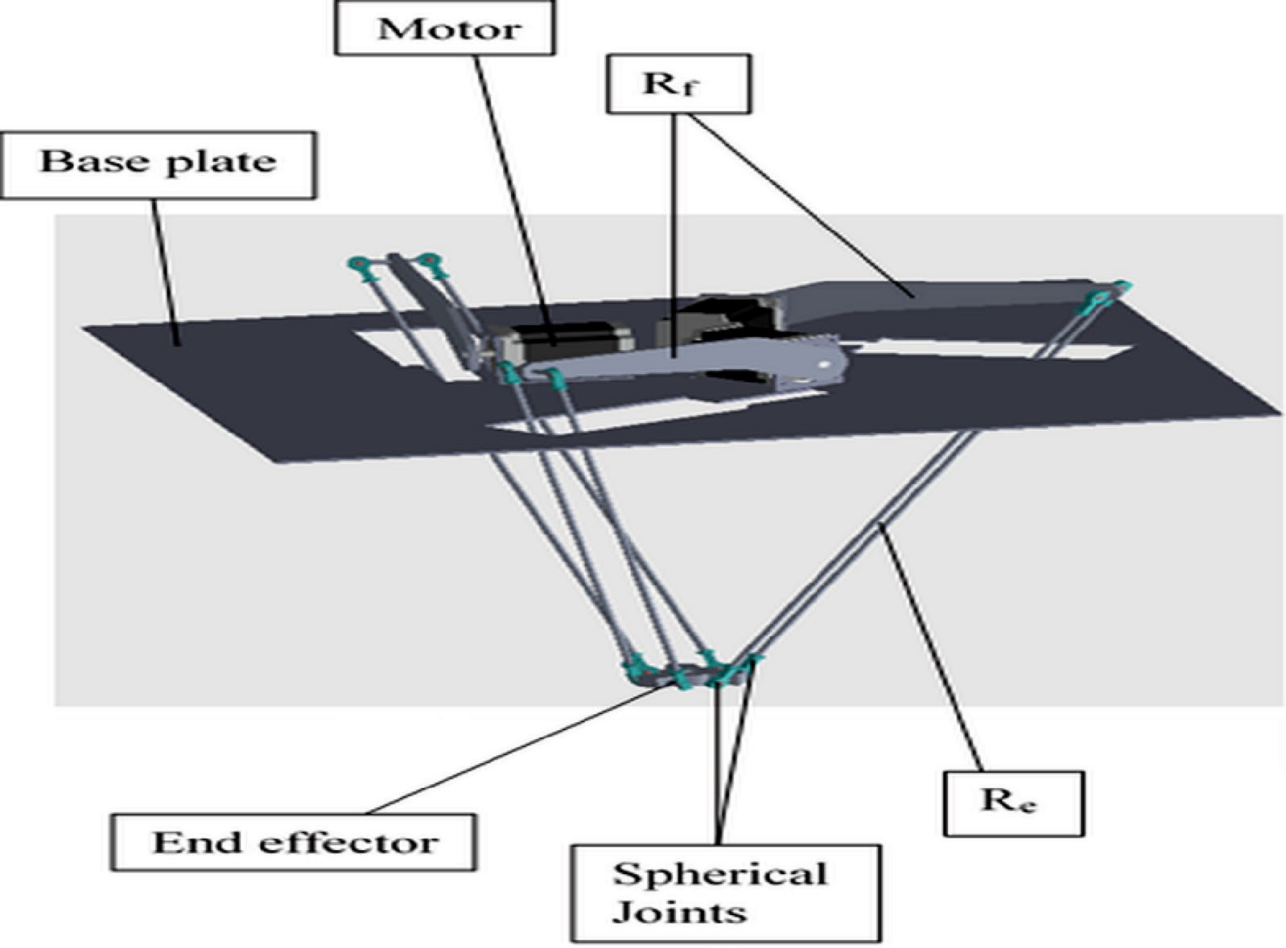
Solidworks model.

Figure 2 illustrates the SolidWorks model of the Delta robot developed using a reverse engineering approach based on the actual system. This model is utilized to improve the mechanical design by minimizing stresses on the links, reducing weight, and lowering overall cost. Additionally, the SolidWorks model serves as a basis for generating a Simscape model, enabling the implementation and comparison of different control strategies to determine the optimal controller for enhanced system performance.

**Fig. 3:**
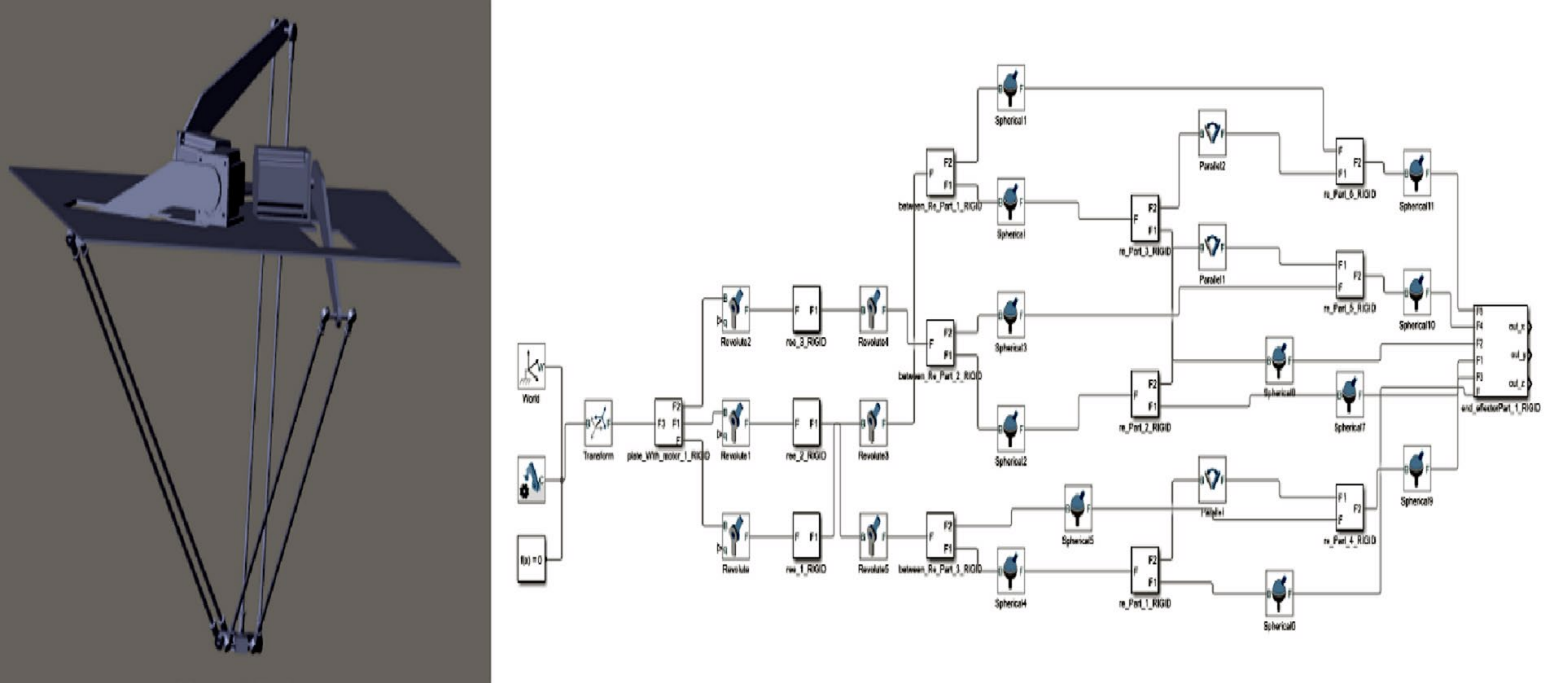
Delta robot simscape model.

After obtaining a validated SolidWorks model of the Delta robot, it is converted into a Simscape model, as shown in Fig. 3, to facilitate dynamic simulation and controller implementation.

**Fig. 4:**
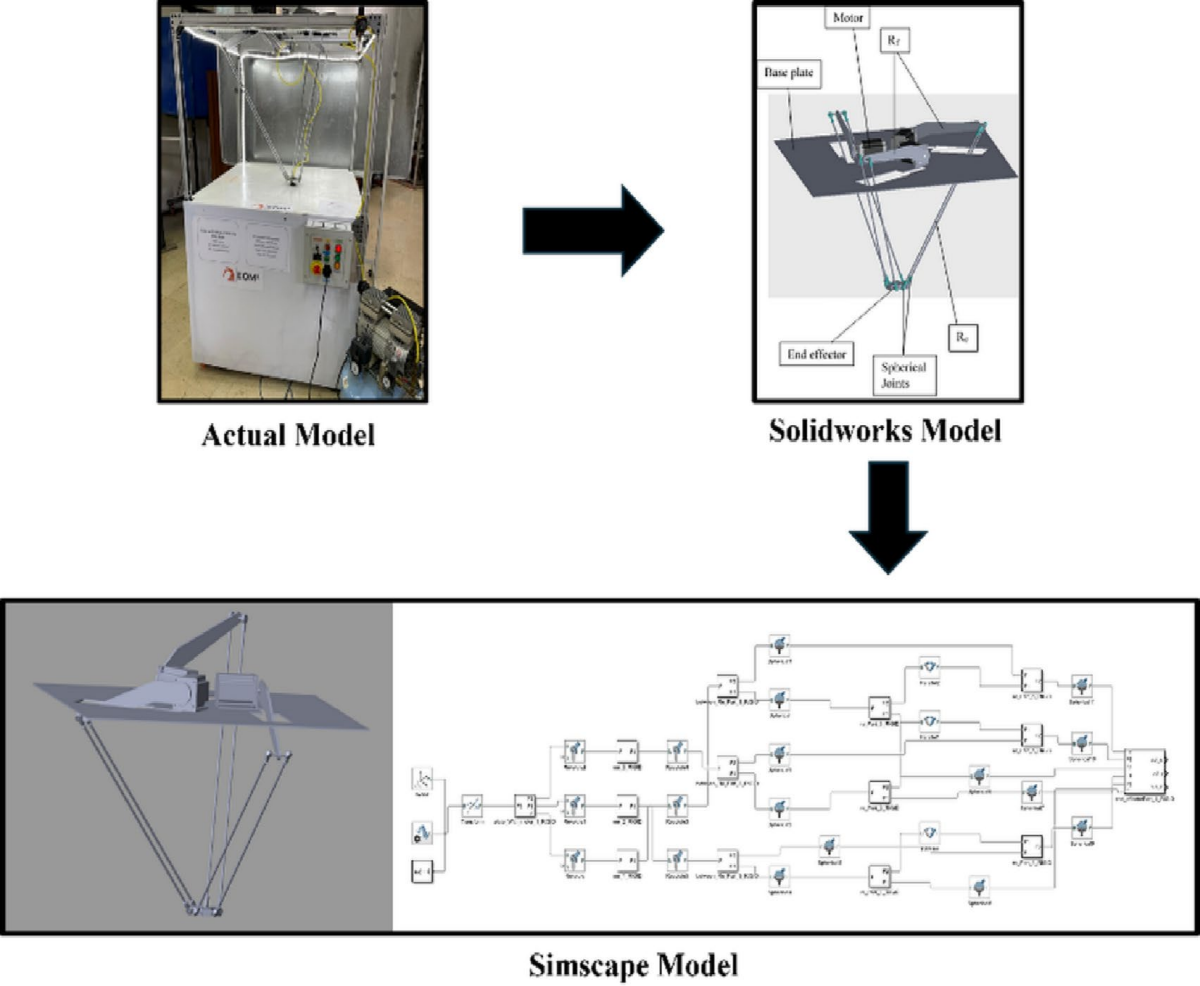
Delta robot system initiation.

The actual hardware system is successfully converted into a Simscape model, enabling the implementation of various control strategies to enhance the overall performance of the Delta robot. Figure 4 illustrates the complete process of developing the Simscape model.

**Fig. 5:**
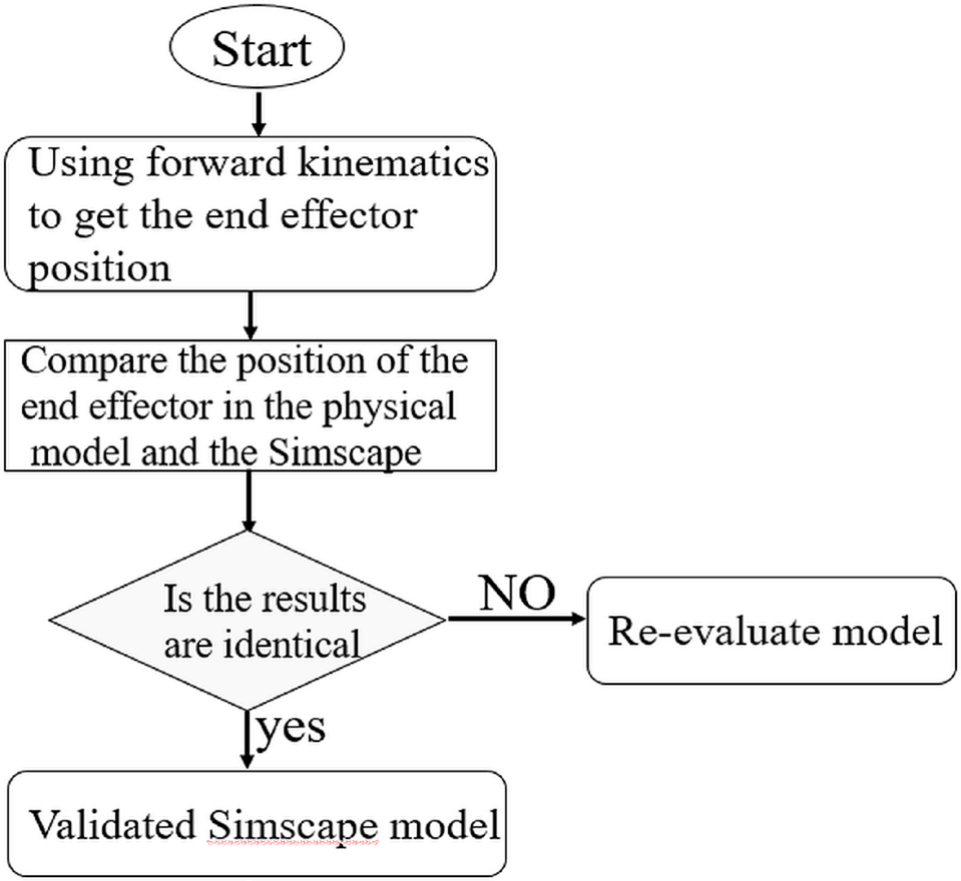
System validation.

The Simscape model is validated to ensure that improvements achieved through control techniques accurately reflect the performance of the real Delta robot system. The validation process follows the steps outlined in the flowchart shown in Fig. 5, resulting in a reliable model suitable for evaluating the proposed control strategies.

### System identification

**Fig. 6:**
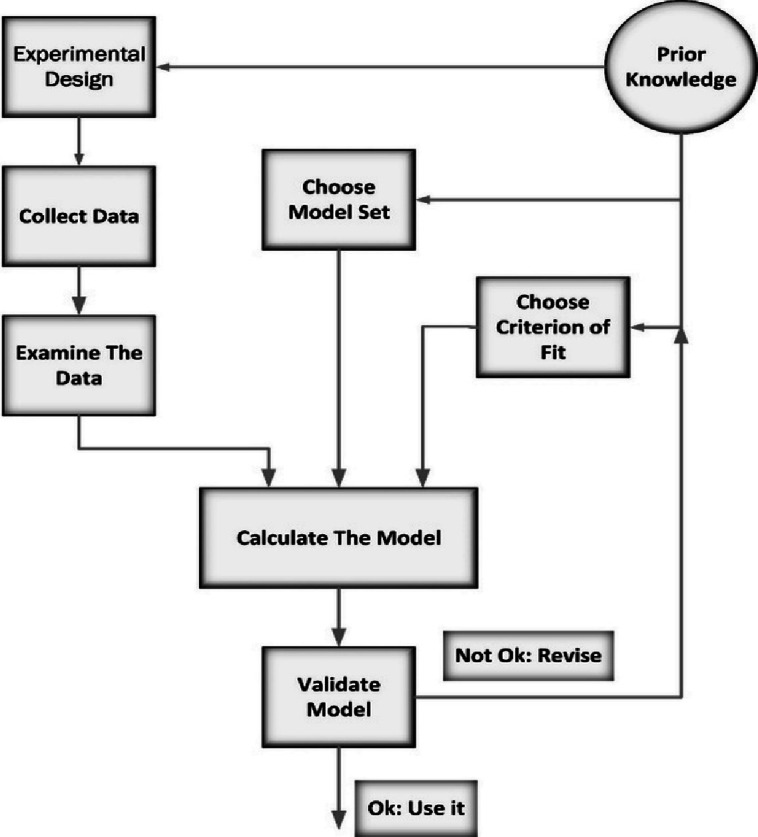
System identification loop.

Because the full Simscape model is computationally expensive, system identification was used to derive a simpler mathematical model from input-output data, as shown in Fig. 6. The process includes experiment design, data collection, preprocessing, model-structure selection, parameter estimation, and validation. Candidate structures may include black-box and gray-box models such as ARX, ARMAX, output-error, Box-Jenkins, and state-space representations. Accurate data collection is essential; therefore, excitation signals should cover the full operating range, the sampling time should be suitable for the robot dynamics, and sensors and data-acquisition hardware should be properly calibrated.

**Fig. 7:**
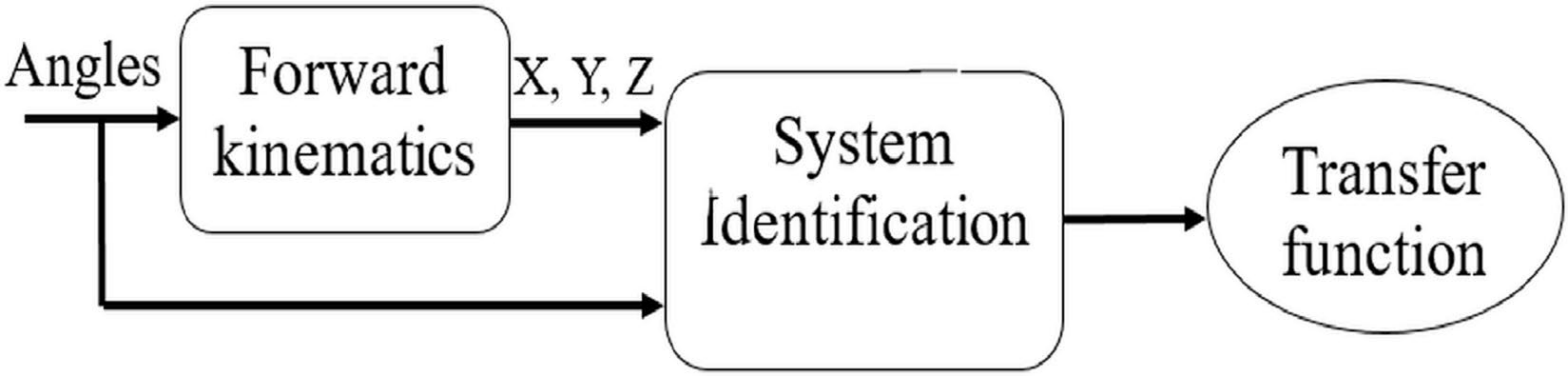
Block diagram for system identification.

MATLAB System Identification Toolbox was used to identify the Delta robot dynamics from multi-input multi-output (MIMO) data. The joint angles were used as inputs, while the end-effector positions were used as outputs. Although both linear and nonlinear least-squares approaches were considered, the nonlinear approach was selected because it better captures the nonlinear behavior of the Delta robot and provides a more accurate model for controller tuning Fig. 7.

**Fig. 8:**
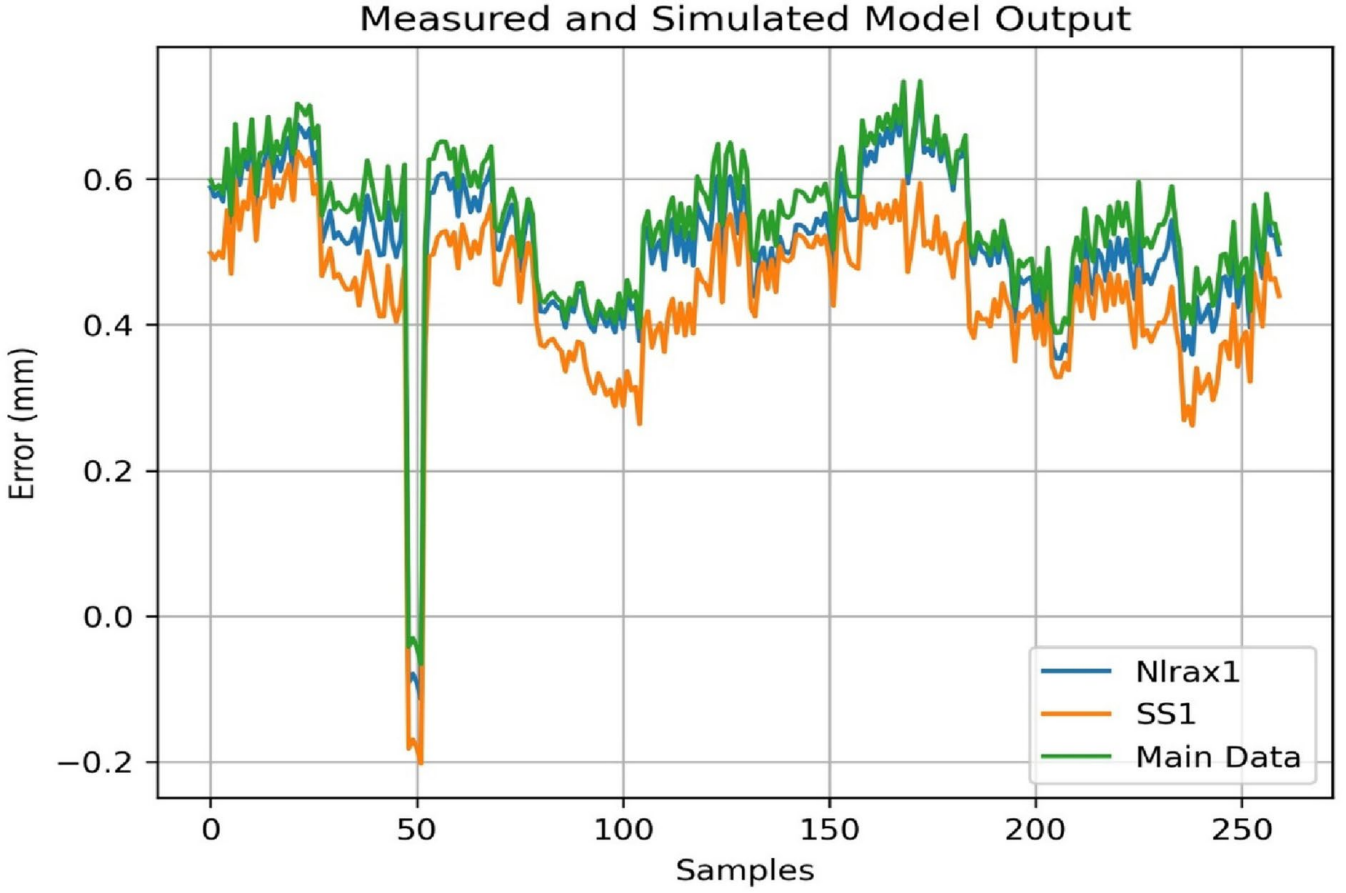
Performance of Nlrax model and actual data.

After obtaining an initial state-space representation in MATLAB, a nonlinear autoregressive model with exogenous inputs (NLARX) was selected to improve the representation of the robot dynamics. The NLARX response closely matched the original Simscape data, as shown in Fig. 8. The identified model achieved a mean square error of 0.02662, confirming that it is sufficiently accurate for subsequent controller tuning and comparative performance analysis.

### Optimization algorithms

- Several optimization methods can be used for controller tuning, including gradient descent, stochastic gradient descent, Newton and quasi-Newton methods, conjugate gradient, genetic algorithms, simulated annealing, particle swarm optimization, ant colony optimization, and Harmony Search. The suitability of each method depends on the problem dimension, search-space limits, nonlinear behavior, and computational cost.
- In this study, such optimization techniques are considered to efficiently tune the controller parameters and achieve improved system performance for the Delta robot control system. Classical tuning methods such as trial-and-error and Ziegler–Nichols are commonly used for controller parameter selection; however, they are often unreliable for achieving optimal performance. Therefore, in this study, the Harmony Search (HS) optimization algorithm is introduced as a more robust and efficient method to determine the optimal parameters of the proposed controllers (PID, NPID, and TID) based on system response and performance requirements.

Harmony Search (HS), proposed by Geem et al. in 2001, is a population-based metaheuristic inspired by musical improvisation. Each decision variable is treated as a musical pitch, and a complete candidate solution is treated as a harmony. The objective function evaluates the quality of each harmony, and improved harmonies are stored in the harmony memory. New solutions are generated by memory consideration, pitch adjustment, and random selection, allowing both global exploration and local refinement of controller parameters.

The initial population of Harmony Memory (HM) is generated randomly. HM consists of Harmony Memory Solution (HMS) vectors where $\:{x}_{i}={x}_{ij},\:i=1,\dots\:\dots\:,HMS\:and\:j=1,\dots\:\dots\:,n.$ The HM is filled with HMS vectors as follows^50^:

1 $$\:HM=\left[\begin{array}{ccccc}{x}_{11}&\:{x}_{12}&\:{x}_{13}&\:\dots\:&\:{x}_{1n}\\\:{x}_{21}&\:{x}_{22}&\:{x}_{23}&\:\dots\:&\:{x}_{2n}\\\:.&\:.&\:.&\:.&\:.\\\:.&\:.&\:.&\:.&\:.\\\:.&\:.&\:.&\:.&\:.\\\:{x}_{HMS1}&\:{x}_{HMS2}&\:{x}_{HMS3}&\:\dots\:&\:{x}_{HMSn}\end{array}\right]$$

Harmony Memory Consideration (HMC) rule, for this rule a new number r1 is generated within the range [0 1]. If r1 < HMCR, where HMCR is the harmony memory consideration rate, then the first decision variable in the new vector $\:{x}_{newij}$ is chosen randomly from the values in the current HM as follows^8^:

2 $$\:{x}_{ij}^{new}={x}_{ij},\:\:{x}_{ij}\in\:\{{x}_{1},\:{x}_{2},{x}_{3},\dots\:\dots\:,{x}_{HMSJ}\}$$

The obtained decision variables from the harmony memory consideration rule is further examined to determine if it needs to pitch adjustment or not. A new random number r2 is generated within the range [0 1]. If r2 < PAR, where PAR is a pitch adjustment rate, then the pitch adjustment decision variable is calculated as follows:

3 $$\:{x}_{ij}^{new}={x}_{ij}\pm\:rand\left(\mathrm{0,1}\right).BW$$

Where BW is a bandwidth factor, which is used to control the local search around the selected decision variable in the new vector. Random initialization rule, If the condition r1 < HMCR fails, the new first decision variable in the new vector $\:{x}_{ij}^{new}$ is generated randomly as follows:

4 $$\:{x}_{ij}^{new}={l}_{ij}+\left({u}_{ij}-{l}_{ij}\right).rand\left(\mathrm{0,1}\right)$$

**Fig. 9:**
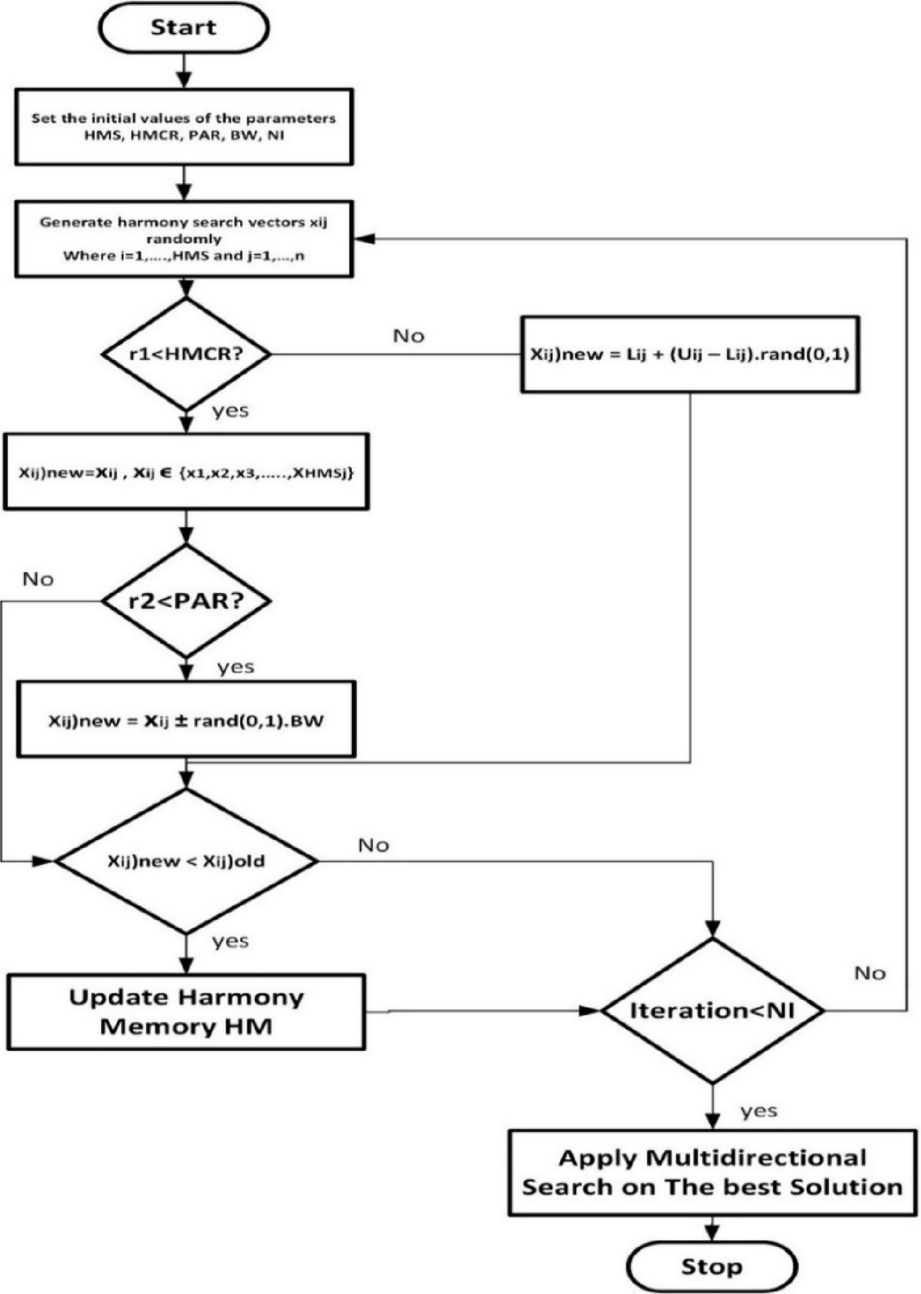
Harmony search tuning system flow chart.

Where l, u is the lower and upper bound for the given problem. After the harmony vector $\:{x}^{\left\{new\right\}}$ is generated, it will replace the worst harmony vector $\:{x}^{\left\{worst\right\}}$ in the harmony memory if its objective function value is better than the objective function value of the worst harmony vector. Figure 9 demonstrates the Harmony search tuning system flow chart.

Finally, in this study, the harmony search optimization technique will be used to obtain the optimal parameters of the TID control and the PID control according to the objective function as shown in Eq. (5).

5 $$\:f=\frac{1}{\left(1-{e}^{-\beta\:}\right)\left({M}_{p}+{e}_{ss}\right)+{e}^{-\beta\:}({t}_{s}-{t}_{r})}$$

Where $\:{M}_{p}$ is the system response overshoot, $\:{e}_{ss}$ is the steady state error, $\:{t}_{s}$ is the settling time and $\:{t}_{r}\:$is the rise time. Further, this objective function can compromise the designer demand based on the weighting parameter value (β). To reduce overshoot and steady-state errors, the parameter is set higher than 0.7. If this parameter is set smaller than 0.7 the rise time and settling time will be reduced.

All candidate parameters are evaluated under the same reference trajectory, simulation duration, actuator saturation, and joint-limit constraints. An infeasible candidate is assigned a penalty and cannot replace a feasible harmony in the harmony memory.

Due to the long computational time required for optimization and the wide range of controller parameters, system identification is adopted as an intermediate step to accelerate the tuning process. This approach enables the extraction of an appropriate parameter range, which is then applied to the original system to further refine the optimization. Initially, forward kinematics is used to generate the required dataset, after which the obtained values are utilized as inputs for the system identification process to develop a simplified and efficient model suitable for controller optimization.

#### Controller parameters and harmony search settings

For reproducibility, the final numerical parameters must be reported exactly as used in the MATLAB/Simulink model. The supplied manuscript did not contain these numerical values; the required entries are therefore identified below for completion from the final model workspace before resubmission Tables 1 and 2.

**Table 1:** Controller parameters, search bounds, initial values, and implemented values.

Controller	Parameters to report	Lower bound(s)	Initial value(s)	Upper bound(s)	Final implemented value(s)
HS-optimized PID	Kp, Ki, Kd	(0.01, 0.01, 0.01)	Randomly initialized within the search space $\:\left[0.01,100\right]$for each controller parameter.	(100, 100, 100)	43.0713,2.9593,9.8256 44.531, 18.3684, 1.5641 49.531, 15.3684, 9.8256
HS-optimized TID	Kt, Ki, Kd, n, Nc	Same as PID	Same as PID	Same as PID	43.0713,2.9593,9.8256 44.531, 18.3684, 1.5641 49.531, 15.3684, 9.8256
Self-tuning fuzzy PID	Baseline Kp, Ki, Kd; Ke, K.Δe, Ku	Not HSO-tuned	Baseline optimized PID gains	7 membership functions (NL, NM, NS, Z, PS, PM, PL) with 49 IF–THEN rules	Fuzzy control signal generated from the rule base using $\:e\:$and $\:{\Delta\:}e$; PID gains remain unchanged
Self-tuning fuzzy TID	Baseline Kt, Ki, Kd, n, Nc; Ke, K.Δe, Ku	Not HSO-tuned	Baseline optimized PID gains	7 membership functions (NL, NM, NS, Z, PS, PM, PL) with 49 IF–THEN rules	A fuzzy control signal generated from the rule base using $\:e\:$and $\:{\Delta\:}e$; TID gains remain unchanged

**Table 2:** Harmony Search settings and optimization constraints.

Setting	Symbol	Value used in the final optimization
Harmony-memory size	HMS	80
Maximum improvisations/iterations	N_iter	10,000,000
Harmony-memory consideration rate	HMCR	0.2
Pitch-adjustment rate	PAR	0.25
Bandwidth or bandwidth schedule	BW	0.8
Objective weighting factor	β	0.7
Random seed and independent runs	—	0.2
Stopping criterion	—	0.01

## Control techniques

**Fig. 10:**
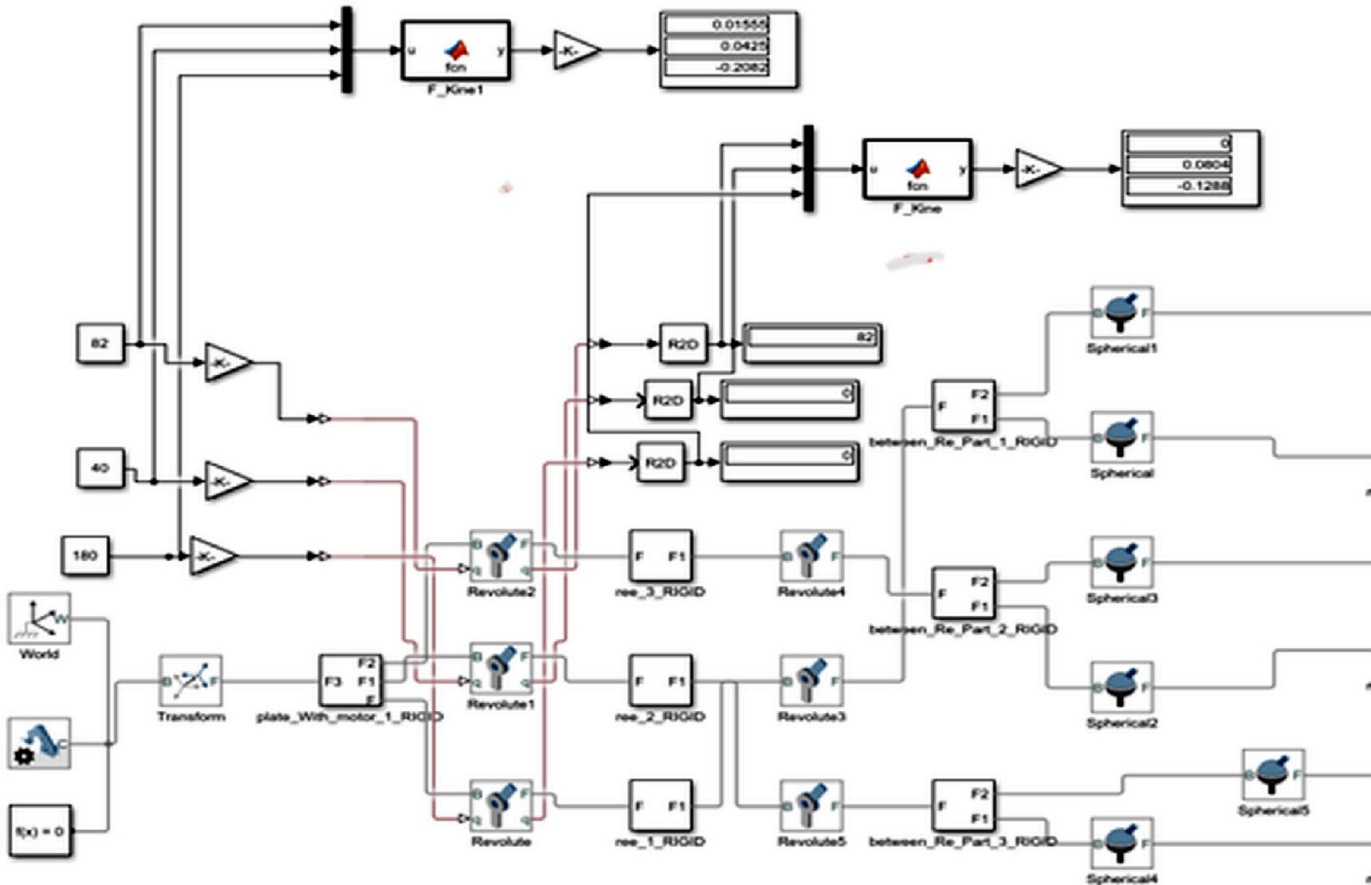
System response without control.

The robot was first tested in open-loop operation to demonstrate the need for feedback control. Arbitrary joint commands of 82° for the first joint, 40° for the second joint, and 180° for the third joint were applied, as shown in Fig. 10. The response showed a clear tracking failure: only the first joint approached the desired position, while the second and third joints remained near 0°. This confirmed that the uncontrolled system has poor tracking accuracy and requires closed-loop control to improve stability and precision.

Accordingly, PID and TID controllers are introduced for the Delta robot, with their parameters tuned using Harmony Search. Fuzzy PID and fuzzy TID are then implemented to improve online adaptation and trajectory-tracking performance. The tests reported in this manuscript evaluate reference tracking; they do not, by themselves, establish rejection of an independently injected external disturbance.

### TID control

**Fig. 11:**
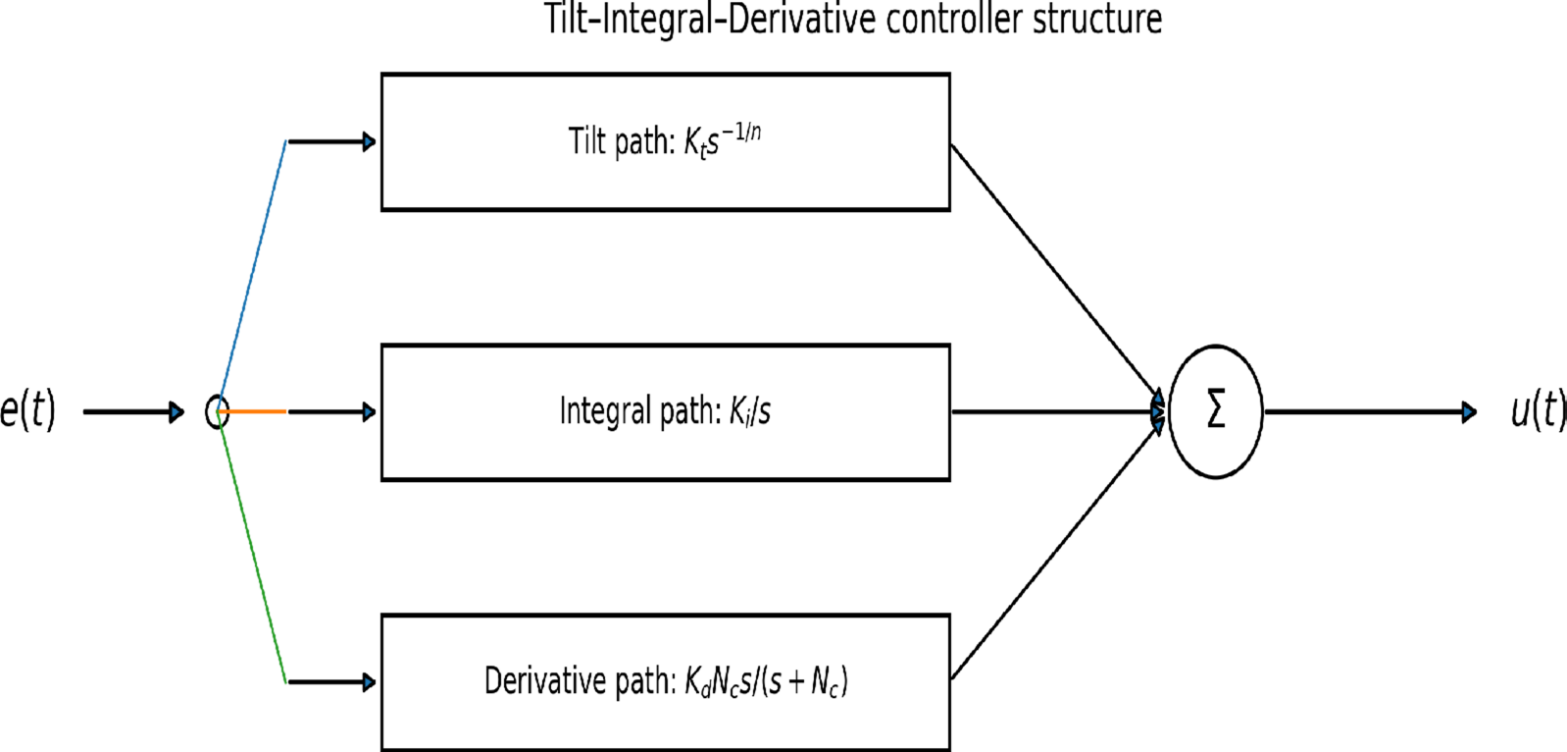
Structure of the TID controller.

PI and PID controllers remain common in industrial servo systems because of their simple structure and low real-time computational burden. The Tilt–Integral–Derivative (TID) controller retains the integral and derivative paths but replaces the constant proportional path of PID with a fractional tilt path. Figure 11 shows the corrected TID structure used in this study.

For reference, the conventional filtered PID controller is C_PID(s) = Kp + Ki/s + Kd. Nc s/(s + Nc).

In the TID controller, K_p is not used. It is replaced by the tilt gain K_t and the fractional term s ^(−1/n)^:

6 $${\rm C_TID(s) = Kt/s^{(1/n)} + Ki/s + Kd \cdot\:Nc s/(s + Nc).}$$

Here Kt is the tilt gain, Ki is the integral gain, Kd is the derivative gain, *n* > 1 determines the fractional tilt order 1/n, and Nc is the derivative-filter coefficient. Thus, Kp denotes the proportional gain only in the PID controller, whereas Kt denotes the tilt gain in the TID and fuzzy-TID controllers.

- The TID controller is particularly useful for dynamic and precision-sensitive systems such as Delta robots. By introducing the tilted action alongside integral and derivative terms, the controller can provide smoother responses and improved tracking behavior compared with a conventional fixed-gain PID controller. However, these benefits require careful parameter selection, which motivates the use of Harmony Search Optimization in this study.
- In contrast to a classical PID controller, the TID controller replaces the proportional path with a tilt term while retaining integral and derivative actions. This additional design parameter improves the ability to shape the closed-loop response, but it also increases tuning complexity. Therefore, an optimization-based tuning strategy is used to identify suitable controller parameters.
- The TID structure provides an additional frequency-shaping degree of freedom and can improve tracking when its parameters are properly tuned. In this manuscript, its performance is evaluated from step, sinusoidal, and repeating-stair tracking. A separate external-disturbance experiment is required before claiming disturbance-rejection robustness.

###  Fuzzy logic control

- Fuzzy control is a rule-based control strategy suitable for complex and nonlinear systems that are difficult to describe using exact mathematical models. Instead of relying only on precise equations, fuzzy control uses degrees of membership and linguistic rules to represent expert knowledge. The controller first fuzzifies the input variables, applies an inference mechanism based on IF-THEN rules, and then defuzzifies the result to generate a crisp control action.
- It operates using fuzzy sets and linguistic rules. Inputs are first converted into fuzzy values through fuzzification, then processed using a rule-based inference mechanism (IF–THEN rules), and finally converted back into a crisp output through defuzzification to produce a real control action.
- The main advantages of fuzzy control are its ability to handle uncertainty without requiring an exact system model, its similarity to human reasoning, and its adaptability to changing system conditions by adjusting rules or membership functions.

#### Fuzzy self-tuning

**Fig. 12:**
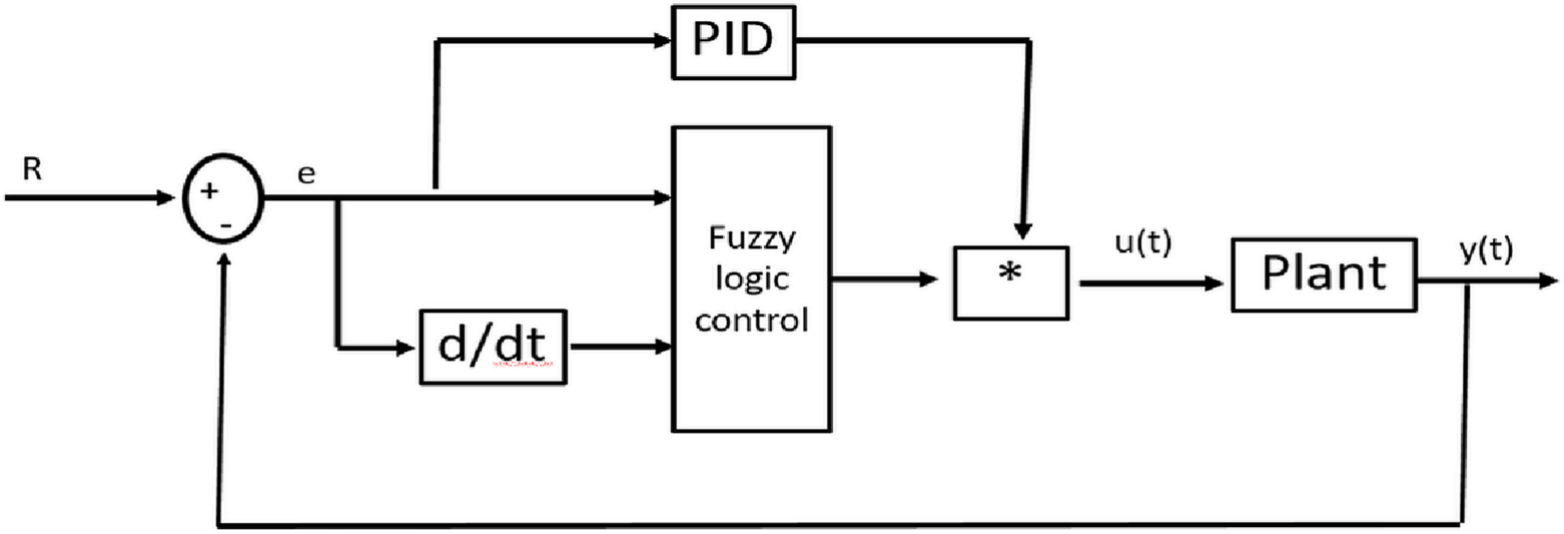
Self-Tunned Fuzzy PID control.

After the system output is brought under closed-loop control, fuzzy self-tuning can be used to adjust the control signal online and reduce tracking error, as shown in Fig. 12. In this architecture, fuzzy logic does not replace the PID or TID controller; instead, it provides an adaptive layer that modifies the controller behavior according to the current error and the change in error. This is particularly useful for the Delta robot because nonlinearities and uncertainties can reduce the effectiveness of fixed controller gains.

**Fig. 13:**
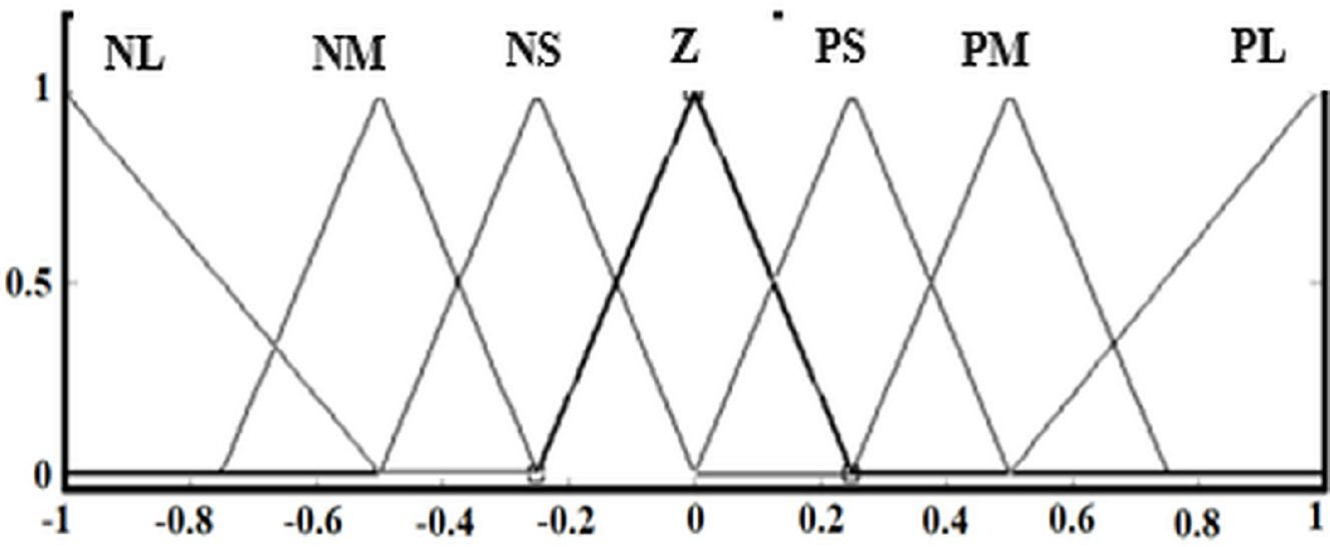
Input membership functions.

**Fig. 14:**
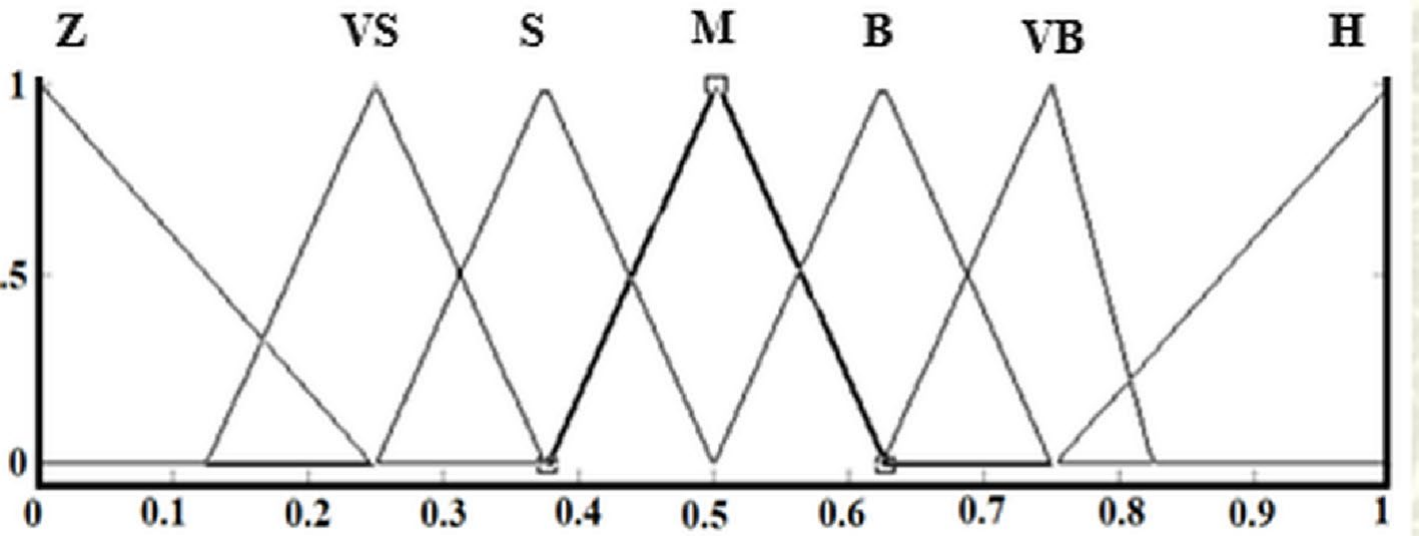
Output membership functions.

In the simulation, the input and output variables are represented by symmetric triangular fuzzy sets, and a rule database consisting of 49 fuzzy rules is used (as shown in Figs. 13 and 14). Table 3 is used to simplify the rule bases. The linguistic labels used for the self-tuning PID controller are as follows: N for negative, P for positive, Z for zero, L for small, A for medium, and G for large. For instance, NA represents negative-medium, PG represents positive-big, and so on.

The normalizing error input gain is represented as Ke, while the gaining error input normalization is represented as K. Next, the normalizing gain values for e and ∆e are computed. The rule bases for determining,, and can be found in^51^, and the defuzzification method utilized is the center of gravity. Further information on this process is provided and analyzed in^51^. The rules used to determine,, and are shown in Table 3.

**Table 3:** Rule base of fuzzy.

∆e E	NL	NM	NS	Z	PS	PM	PL
NL	H	H	H	H	H	H	H
NM	VB	VB	VB	VB	B	VB	H
NS	B	B	B	B	VB	B	H
Z	Z	Z	Z	VS	S	S	S
PS	B	B	B	B	VB	B	H
PM	VB	VB	VB	VB	VB	VB	H
PL	H	H	H	H	H	H	H

As a last step, apply the defuzzification procedure to convert the fuzzy output into crisp values that may be used as non-fuzzy control actions.The most widely used center-of-gravity or center-of-area defuzzification technique, which is formulated as follows:

7 $$\:U=\:\frac{\sum\:_{i=1}^{r}u\left({u}_{i}\right){u}_{i}}{\sum\:_{i=1}^{r}u\left({u}_{i}\right)}$$

Where: u($\:{u}_{i}$) is the element’s membership grad (weight) and $\:{u}_{i}$ is the output of the rule i.

#### Self-tuning fuzzy PID

Integrating fuzzy logic with PID control overcomes several limitations of conventional PID controllers when applied to complex systems such as Delta robots. Due to their high-speed operation and nonlinear dynamics, Delta robots require adaptive control strategies capable of handling rapid system variations. Traditional PID controllers use fixed gains, which can lead to performance degradation under disturbances, resulting in overshoot, oscillations, and increased settling time.

**Fig. 15:**
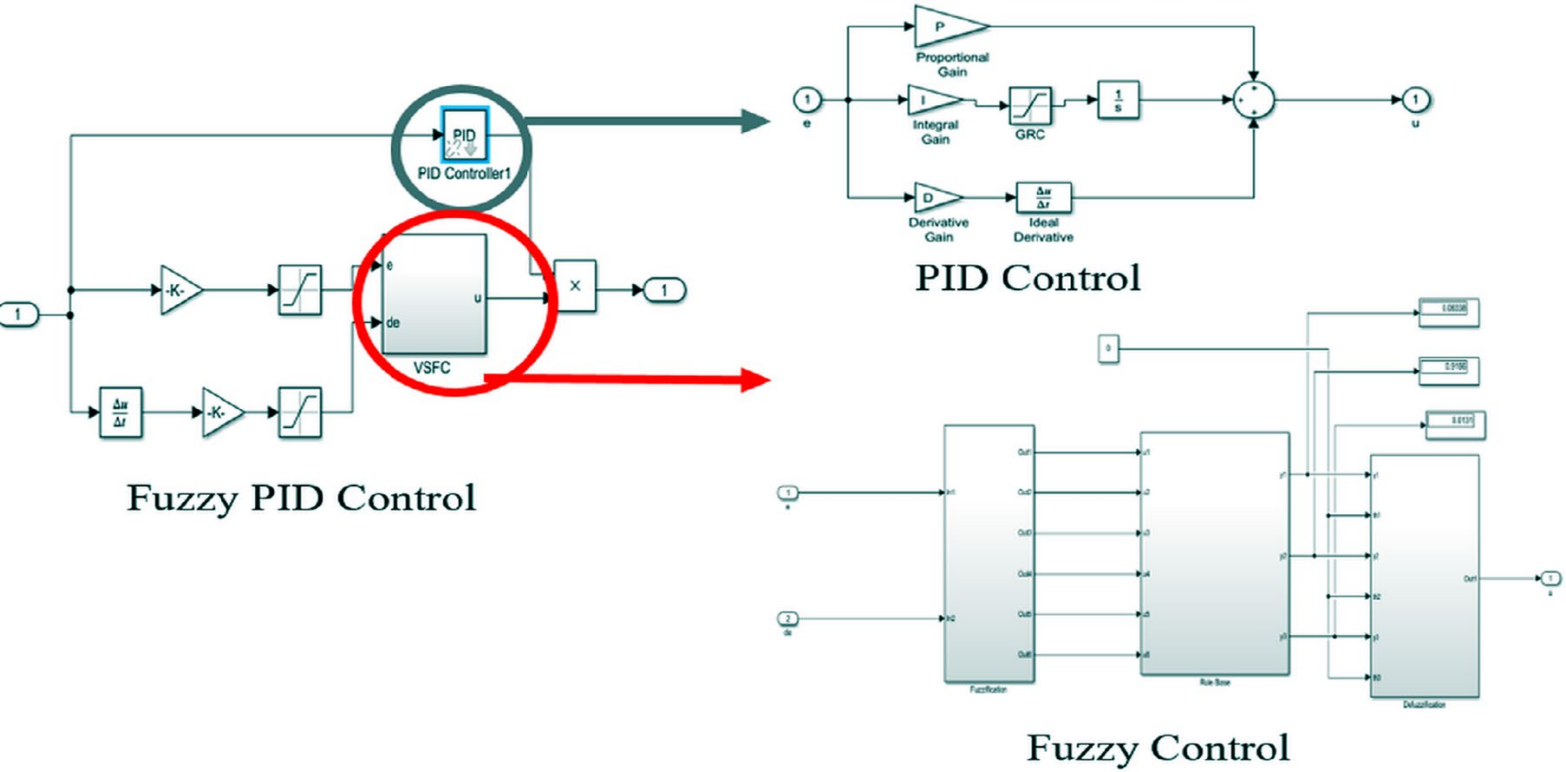
Fuzzy PID Structure in Simscape.

By incorporating fuzzy logic, the controller gains real-time adaptability, allowing the control action to vary with the instantaneous error and change of error. This can reduce overshoot and oscillation under changing references. Because no independent disturbance was injected in the supplied simulations, the present results support improved tracking adaptability rather than a general claim of external-disturbance robustness. The Simscape implementation of the fuzzy PID controller is shown in Fig. 15.

####  Self-tuning fuzzy TID

**Fig. 16:**
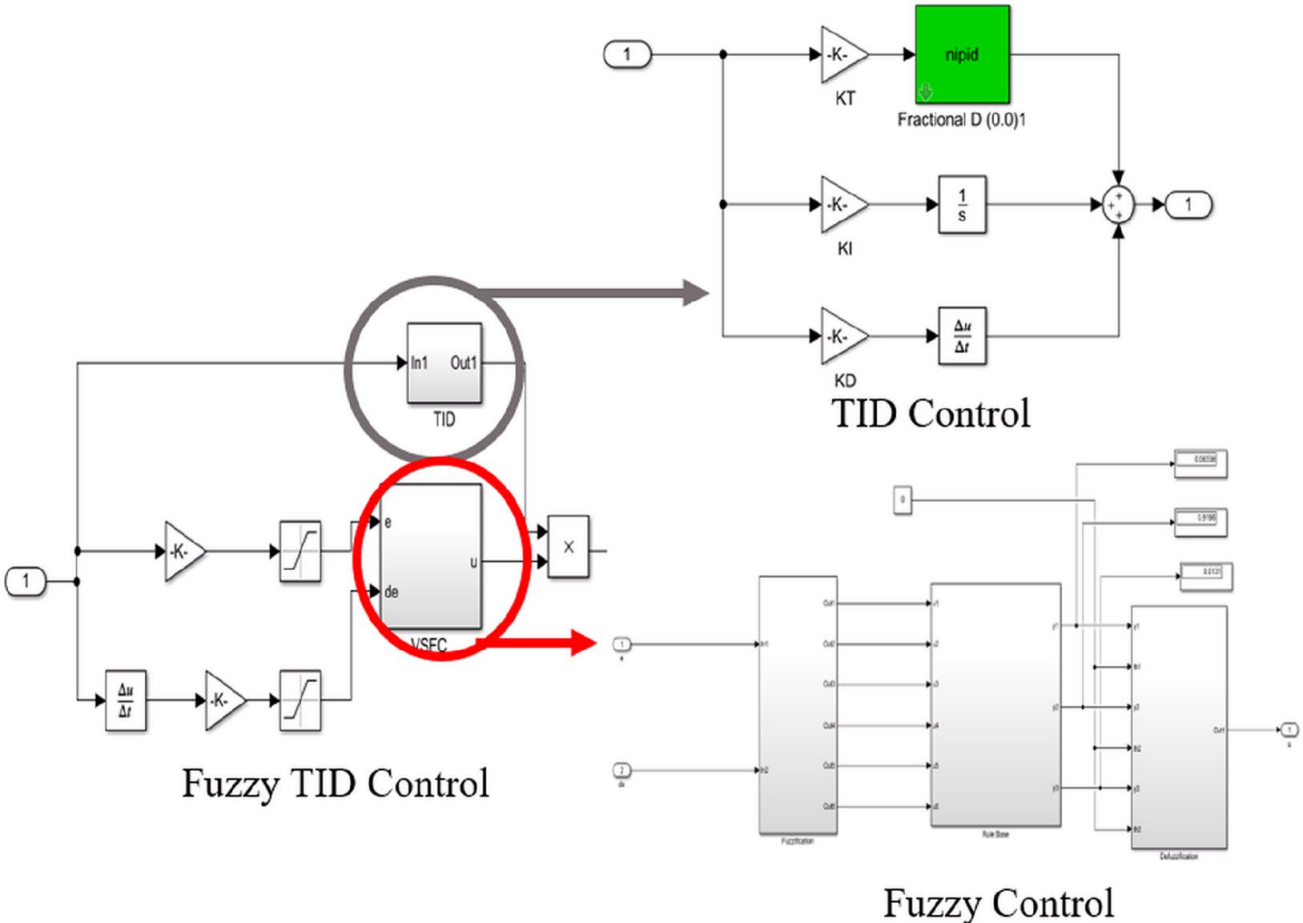
Fuzzy TID structure in Simscape.

In this study, fuzzy logic is integrated with the TID controller to enhance adaptation to the nonlinear and time-varying tracking conditions of the Delta robot. The comparison with fixed-parameter TID evaluates whether the fuzzy layer improves precision, smoothness, and tracking accuracy. External-disturbance robustness is considered a separate validation requirement. Figure 16 shows the Simscape structure of the fuzzy TID controller.

## Results and Discussion

This section presents a comparative analysis of four control strategies—HS-optimized PID, HS-optimized TID, Self-tuning Fuzzy PID, and the proposed Self-tuning Fuzzy TID—to identify the most effective approach for Delta robot control. The evaluation is conducted using three standard test inputs: step, sinusoidal, and repeating stair signals. These tests assess controller performance in terms of stability, tracking accuracy, error reduction, and adaptability to different operating conditions. The results are used to highlight the advantages and limitations of each method and determine the best-performing control strategy.

### Step input test

By using step input, the strengths and weaknesses of each controller can be identified clearly, enabling informed decisions for applications requiring precision, stability, and rapid response in delta robot operations. Step input signals are used to evaluate delta robot joint controllers because they provide a clear way to assess performance under sudden changes. They enable the analysis of transient response characteristics like rise time, overshoot, and settling time, while testing the stability of the controller during abrupt transitions. Step inputs also help detect overshoot, oscillations, and robustness in handling rapid changes, making them ideal for tuning controller parameters.

**Fig. 17:**
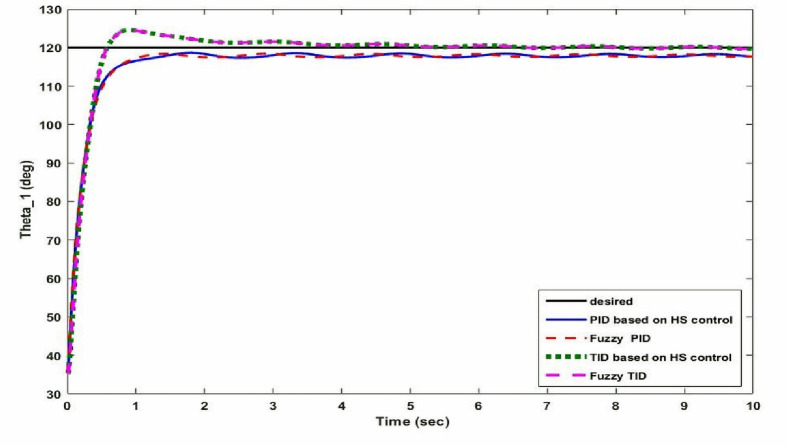
Comparison for theta_1 response between PID &TID Based on HS, Fuzzy PID &Fuzzy TID for Step Input.

Figure 17 presents a comparison of the responses of the PID, TID, Fuzzy PID, and Fuzzy TID controllers for the first joint of the Delta robot (θ₁) tracking a desired angle of 120°. The PID and TID controllers show minor oscillations around the reference value, whereas the Fuzzy PID and Fuzzy TID controllers exhibit smoother and more stable responses with reduced oscillations. Among all tested methods, the Fuzzy TID controller provides the best performance, achieving the closest tracking to the desired angle and demonstrating superior precision and stability.

All controllers closely track the desired trajectory, with only small deviations observed during both transient and steady-state responses. However, the Fuzzy PID and Fuzzy TID controllers demonstrate superior stability, exhibiting fewer oscillations during the settling phase compared to the PID and TID controllers. Although the settling time is generally comparable across all methods, the Fuzzy TID achieves smoother convergence and faster stabilization around the steady-state value. In contrast, the PID and TID controllers show slightly higher oscillatory behavior during transient response. Overall, the Fuzzy TID controller provides the best performance in terms of fast settling, reduced oscillations, and improved trajectory tracking accuracy, making it the most suitable for high-precision applications. The performance metrics for each controller are summarized in Table 4.

**Table 4:** Performance Comparison for theta_1 between PID &TID Based on HS, Fuzzy PID &Fuzzy TID.

Rise time	PID	TID	Fuzzy PID	Fuzzy TID
	0.4697	0.7020	0.8892	0.7012
Settling time	0.8395	3.8456	1.3413	3.8403
Overshoot %	0.5449	4.0929	0.5696	4.0914
Steady state error	2.2705	0.2982	2.2705	0.3022

**Fig. 18:**
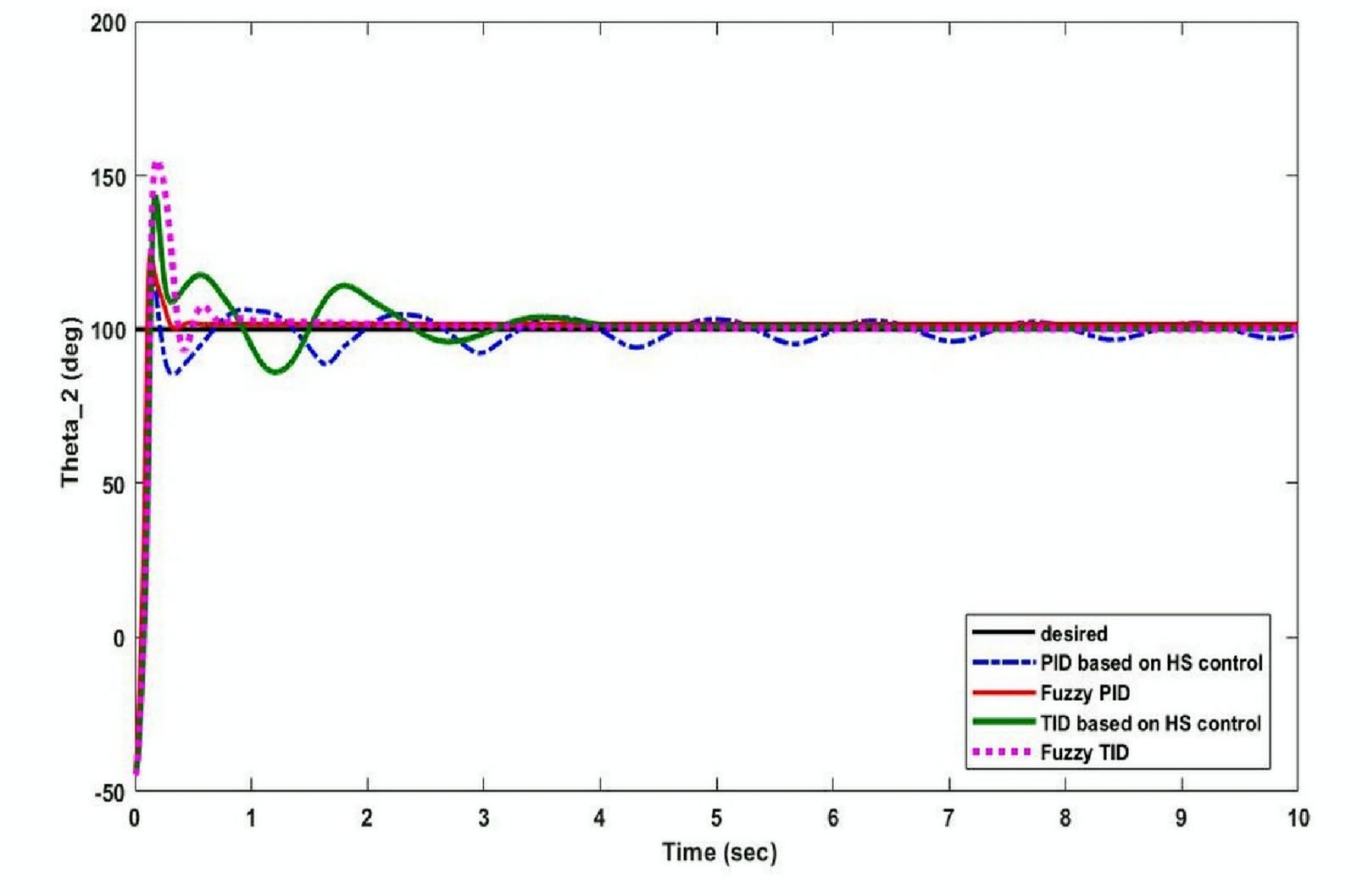
Comparison for theta_2 response between PID & TID Based on HS, Fuzzy PID &Fuzzy TID for Step Input.

Figure 18 presents a comparison of the responses of the PID, TID, Fuzzy PID, and Fuzzy TID controllers for the second joint of the Delta robot (θ₂) tracking a desired angle of 100°. The PID and TID controllers show noticeable oscillations around the reference value, whereas the Fuzzy PID and Fuzzy TID controllers exhibit smoother and more stable responses. Among all methods, the Fuzzy TID controller achieves the best performance, with minimal oscillations, improved stabilization, and closer tracking to the desired trajectory, resulting in higher precision and overall system stability.

All controllers demonstrate good tracking of the desired trajectory with minimal steady-state error. However, more pronounced initial deviations are observed in the HS-optimized TID controller. Both Fuzzy PID and Fuzzy TID controllers exhibit improved stability, with reduced oscillations during the settling phase, whereas the TID controller shows relatively higher oscillatory behavior before convergence. Although the settling time is comparable across all methods, the Fuzzy TID provides smoother and more stable transitions to the steady-state value. In contrast, the PID and TID controllers present noticeable oscillations, particularly during the initial response, while the fuzzy-based controllers significantly mitigate these effects. Overall, the Fuzzy TID controller delivers the best performance, achieving enhanced stability, smooth response, and minimal oscillations, making it well-suited for high-precision and reliable control applications. The performance metrics for each controller are summarized in Table 5.

**Table 5:** Performance Comparison for theta_2 between PID & TID Based on HS, Fuzzy PID &Fuzzy TID.

	PID	TID	Fuzzy PID	Fuzzy TID
Rise time	0.0747	0.1948	0.2168	0.2188
Settling time	0.2899	4.2741	0.6347	1.9102
Overshoot %	11.9365	43.5529	23.0406	55.0193
Steady state error	1.6640	0.0911	1.6645	0.0770

#### θ₃ Response for step input

**Fig. 19:**
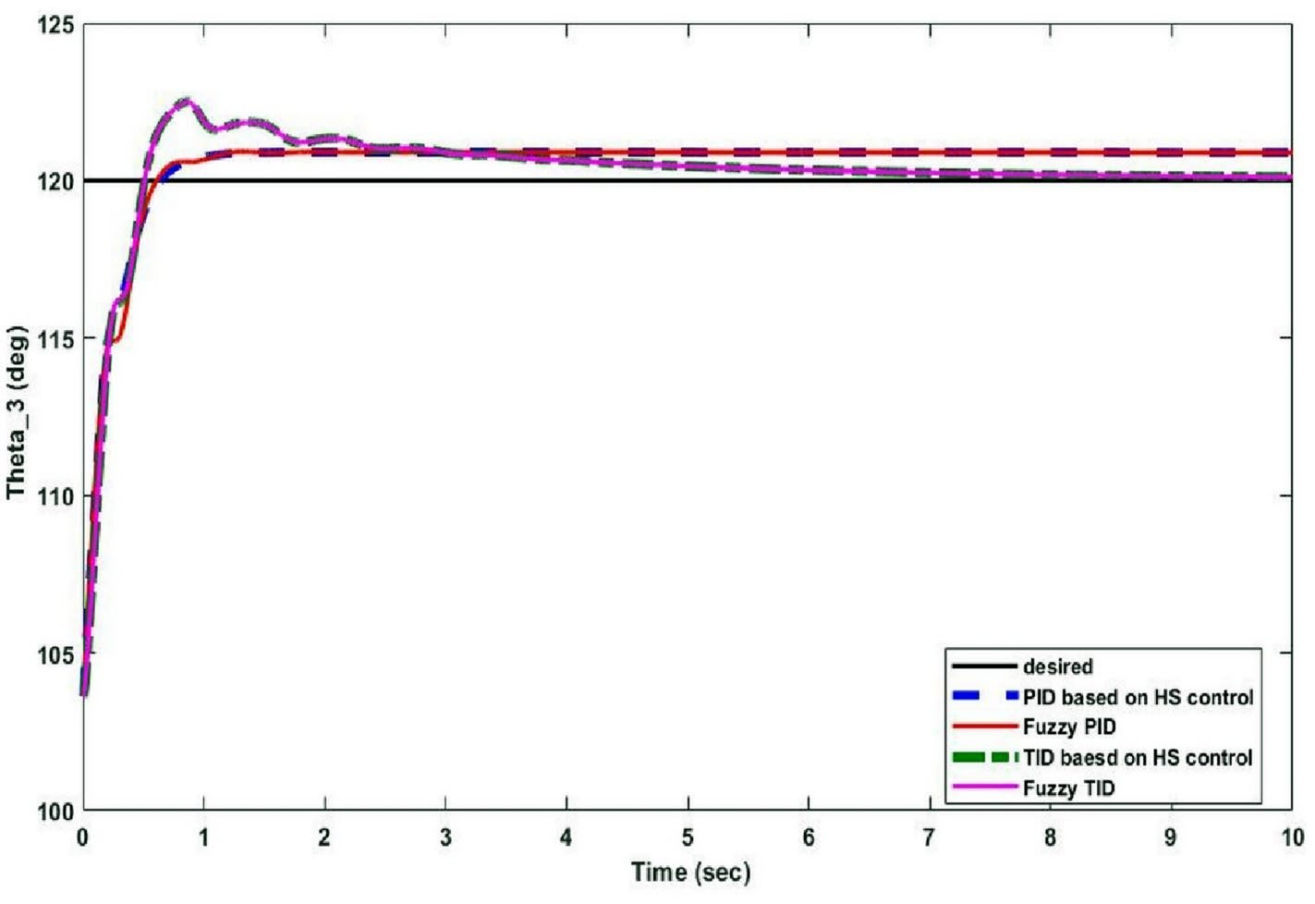
Comparison for theta_3 response between PID & TID Based on HS, Fuzzy PID &Fuzzy TID for Step Input.

Figure 19 presents a comparison of the responses of the PID, TID, Fuzzy PID, and Fuzzy TID controllers for the third joint of the Delta robot (θ₃) tracking a desired angle of 120°. The PID and TID controllers exhibit minor oscillations around the reference value, whereas the Fuzzy PID and Fuzzy TID controllers show smoother and more stable responses. Among all controllers, the Fuzzy TID achieves the best performance, with reduced oscillations, faster stabilization, and improved tracking accuracy, making it the most effective approach for precise and stable control.

All controllers achieve close tracking of the desired trajectory with minimal deviations; however, the PID and TID controllers exhibit slight overshoot during the initial response. In contrast, the Fuzzy PID and Fuzzy TID controllers demonstrate improved stability, with smoother transitions and reduced oscillations during the settling phase, while the TID controller shows more pronounced oscillatory behavior at the beginning of the response. Although the settling times are generally comparable across all controllers, the fuzzy-based methods reach steady behavior more quickly and maintain it more effectively. Additionally, oscillations observed in the PID and TID controllers during settling are significantly reduced by the fuzzy controllers. Overall, the Fuzzy TID controller achieves the best performance in terms of stability, smooth response, and minimal overshoot, making it the most suitable option for high-precision applications. The performance metrics for each controller are summarized in Table 6.

**Table 6:** Performance Comparison for theta_3 between PID & TID Based on HS, Fuzzy PID &Fuzzy TID.

	PID	TID	Fuzzy PID	Fuzzy TID
Rise time	0.4916	0.6595	0.8691	0.6595
Settling time	0.7469	5.9806	1.3455	5.9781
Overshoot %	0.0256	1.9941	0.0244	1.9869
Steady state error	0.8889	0.1152	0.8892	0.1149

### Sine-wave input test

Sine wave inputs are used to evaluate Delta robot joint controllers because they represent smooth and continuous variations similar to real-world motion trajectories. They enable assessment of the controller’s ability to accurately track dynamic reference signals under changing conditions, providing insight into stability and precision. This type of input is particularly useful for analyzing the controller’s response to periodic variations in frequency and amplitude. Additionally, sine wave testing highlights key performance aspects such as tracking accuracy, robustness, and oscillatory behavior during continuous motion, making it an effective benchmark for evaluating control performance in precision-demanding applications.

**Fig. 20:**
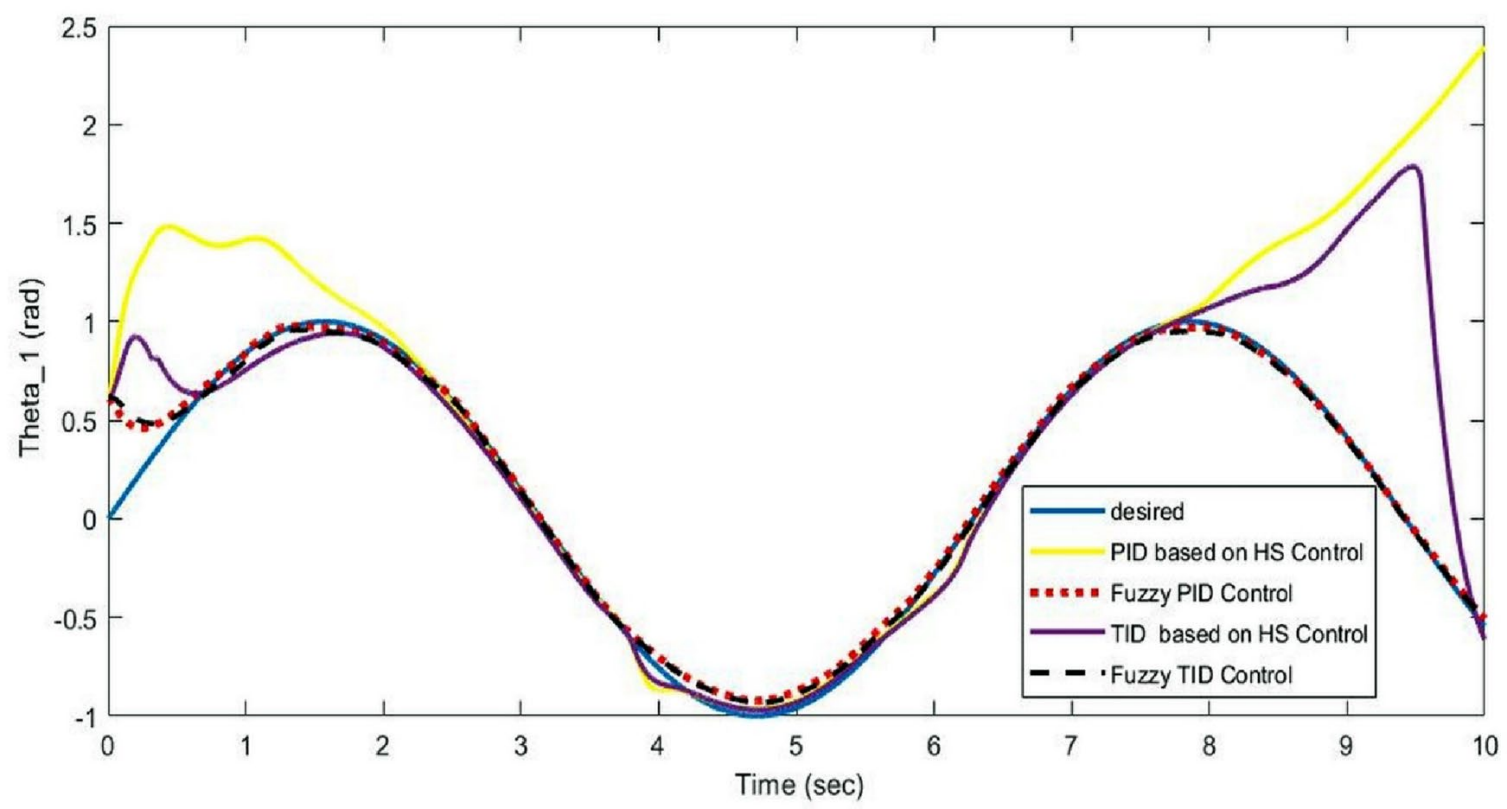
Comparison for theta_1 response between PID &TID Based on HS, Fuzzy PID &Fuzzy TID for Sine Input.

Figure 20 compares the HS-optimized PID, HS-optimized TID, fuzzy PID, and fuzzy TID responses of the first Delta-robot joint (θ₁) under sinusoidal reference motion. PID and TID show more visible oscillation and phase error near transitions, whereas the fuzzy controllers produce smoother dynamic tracking. Among the evaluated cases, fuzzy TID gives the closest reference tracking and the smallest Cartesian RMSE in the X direction.

Across all time phases (0–10 s), the controllers show different tracking performances for θ₁ under sine wave input. The HS-optimized PID exhibits slower response with lag and tracking errors, while the HS-optimized TID responds faster but suffers from overshoot and oscillations. The Fuzzy PID improves stability and reduces deviations, though minor errors remain. Overall, the Fuzzy TID controller consistently provides the best performance, achieving the smoothest response, minimal overshoot, fastest stabilization, and highest tracking accuracy throughout all phases. The comparison is summarized in Table 7.

**Table 7:** Summary of comparison between PID&TID based on HS, Fuzzy PID and Fuzzy TID control of theta_1 performance for sine input.

	PID Control	TID Control	Fuzzy PID Control	Fuzzy TID Control
Response Smoothness	Exhibits significant oscillations, especially at transitions and peak points.	Shows moderate smoothness but with noticeable abrupt changes.	Delivers smoother transitions with reduced oscillations.	Provides the smoothest transitions among all controllers, with minimal oscillatory behavior.
Stability	Moderate stability, struggles to stay aligned with the desired trajectory at peaks and transitions.	Improved stability compared to PID but still has deviations at rapid changes.	High stability, closely aligns with the desired trajectory in most regions.	Very high stability, with consistent to the desired.
Oscillation Control	Exhibits frequent and significant oscillations after transitions.	Reduces oscillations compared to PID but still present at certain points.	Effectively minimizes oscillations, achieving a steadier response.	Nearly eliminates oscillations, ensuring a steady and controlled response.
Settling Pattern	Takes longer time to stabilize, with noticeable overshoot and oscillations.	Stabilizes faster than PID but with minor overshoot.	Settles quickly with minimal overshoot, aligning well with the desired trajectory.	Achieves the quickest and most accurate settling pattern, with minimal overshoot and strong alignment to the desired.

**Fig. 21:**
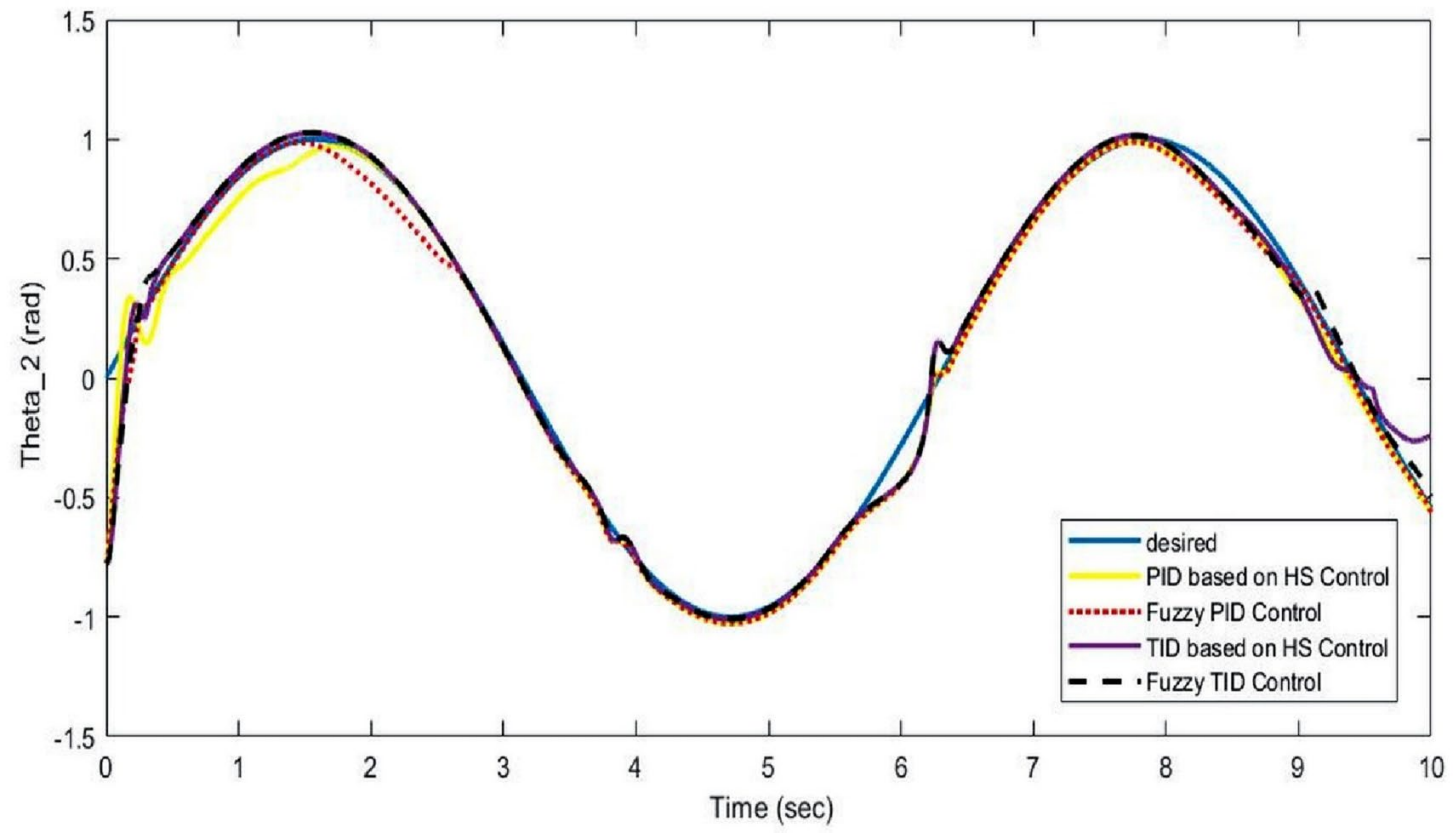
Comparison for θ₂ response among HS-optimized PID, HS-optimized TID, Fuzzy PID, and Fuzzy TID controllers for sine input.

Figure 21 presents a comparison of HS-optimized PID, HS-optimized TID, Fuzzy PID, and Fuzzy TID controllers for the second joint of the Delta robot (θ₂) under a sine wave input. The PID and TID controllers show noticeable oscillations and overshoot during transitions, whereas the Fuzzy PID and Fuzzy TID controllers exhibit smoother and more stable responses with reduced oscillations. Among all methods, the Fuzzy TID controller achieves the best tracking performance, with improved stability and higher accuracy in following the desired trajectory.

Across all phases (0–10 s), the controllers show varying tracking performance for θ₂ under sine wave input. The HS-optimized PID exhibits delay, overshoot, and oscillations, while the HS-optimized TID improves response speed but still suffers from instability at peak transitions. The Fuzzy PID reduces oscillations and improves tracking accuracy, though slight phase lag remains. Overall, the Fuzzy TID controller consistently achieves the best performance, providing the smoothest response, minimal overshoot, fastest stabilization, and highest tracking accuracy throughout all phases. The comparative results are summarized in Table 8.

**Table 8:** Summary of comparison between PID&TID based on HS, Fuzzy PID and Fuzzy TID control of theta_2 performance for sine input.

	PID control	TID control	Fuzzy PID control	Fuzzy TID control
Response Smoothness	Displays significant oscillations, especially during transitions and peaks.	Shows moderate smoothness with sudden changes at transitions.	Achieves smoother transitions and reduced oscillations.	Provides the smoothest response, with minimal oscillatory behavior during transitions and steady-state periods
Stability	Moderate stability, struggles to maintain consistency during rapid transitions and peak points.	Improved stability over PID but still experiences noticeable deviations at transitions.	High stability, maintaining alignment with the desired through most of the response.	Very high stability, with precise adherence to the desired even during rapid transitions.
Oscillation Control	Frequent and significant oscillations, particularly after rapid changes.	Reduces oscillations compared to PID but does not fully eliminate them.	Effectively controls oscillations, resulting in a steadier response.	Nearly eliminates oscillations, achieving the steadiest response among all controllers.
Settling Pattern	Takes longer time to stabilize, with visible overshoot and oscillations.	Stabilizes faster than PID with minor overshoot.	Settles quickly with minimal overshoot and good alignment to the desired trajectory.	Achieves the fastest and most accurate settling pattern, with minimal overshoot and consistent tracking of the desired trajectory.

**Fig. 22:**
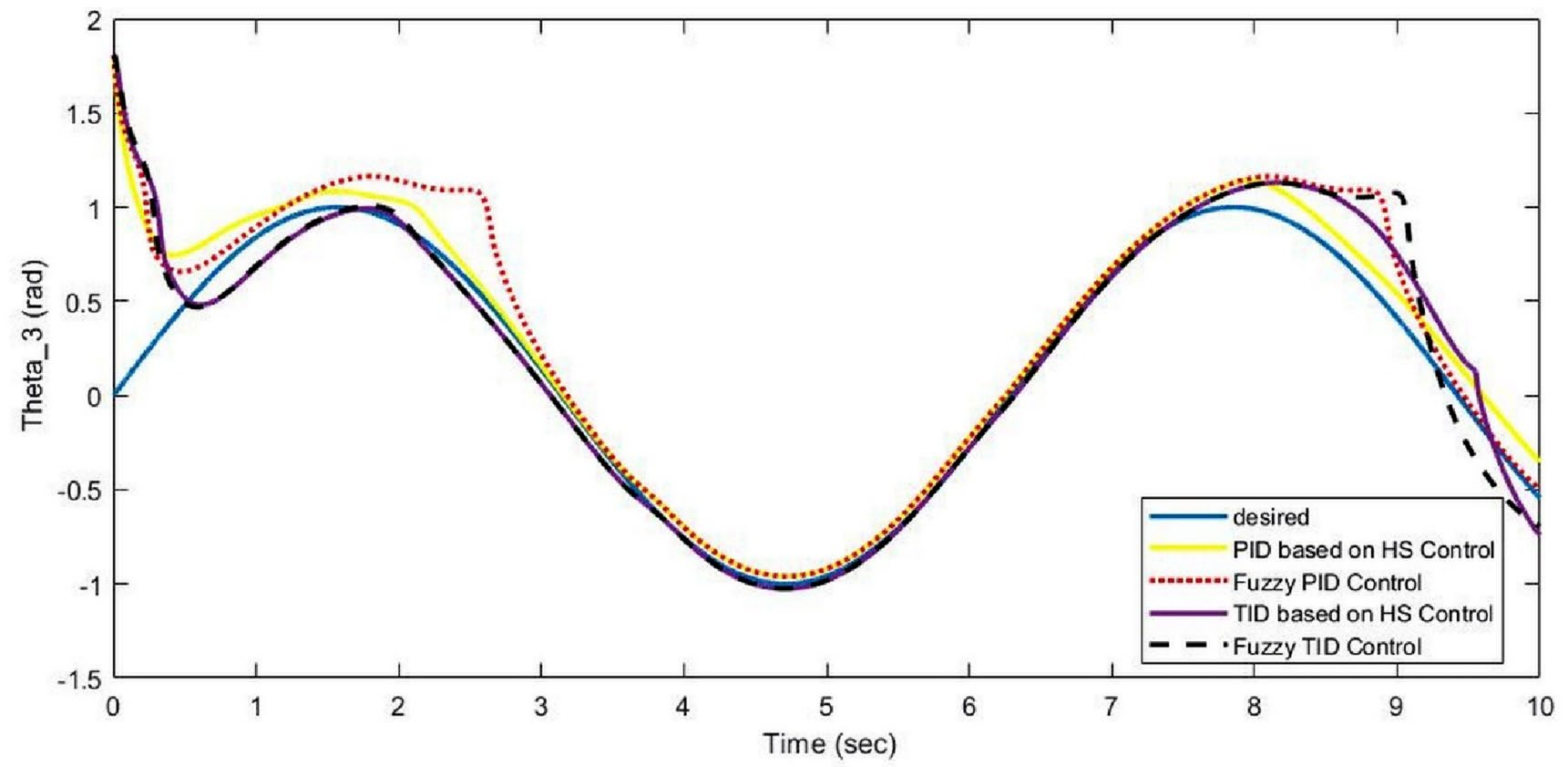
Comparison for theta_3 response between PID &TID Based on HS, Fuzzy PID &Fuzzy TID for Sine Input.

Figure 22 presents a comparison of HS-optimized PID, HS-optimized TID, Fuzzy PID, and Fuzzy TID controllers for the third joint of the Delta robot (θ₃) under sinusoidal input conditions. All controllers are able to track the desired trajectory; however, the Fuzzy PID and Fuzzy TID controllers exhibit smoother responses with reduced oscillations compared to the others. The PID and TID controllers show noticeable overshoot and oscillatory behavior, particularly during transitions. Among all methods, the Fuzzy TID controller achieves the best performance, providing the closest tracking to the reference trajectory with minimal deviations, making it the most suitable for high-precision and stable control applications.

Across all phases (0–10 s), the controllers show different tracking performances for θ₃ under sinusoidal input. In the initial phase, the PID and TID controllers exhibit slower response, overshoot, and oscillations, while the Fuzzy PID improves tracking but still shows minor deviations. The Fuzzy TID provides the best initial behavior with minimal overshoot and smooth convergence. During the middle phase, fuzzy-based controllers maintain better stability and accuracy, whereas PID and TID show phase lag and higher oscillations. In the final phase, PID and TID performance degrades further with noticeable instability, while Fuzzy PID remains moderately stable. Overall, the Fuzzy TID controller consistently achieves the best performance across all phases, delivering smoother response, higher accuracy, and superior stability. The comparative results are summarized in Table 9.

**Table 9:** Summary of comparison between PID&TID based on HS, Fuzzy PID and Fuzzy TID control of theta_3 performance for sine input.

	PID Control	TID Control	Fuzzy PID Control	Fuzzy TID Control
Response Smoothness	Displays significant oscillations, especially during rapid changes and peaks.	Transitions are moderately smooth with a little quickness.	Provides smoother transitions with fewer oscillations.	Achieves the smoothest response, with minimal oscillations and a consistent tracking pattern.
Stability	Moderate stability, struggles with maintaining alignment during rapid transitions.	Improved stability compared to PID but still insights small deviations.	High stability, with consistent alignment to the desired.	Very high stability, maintaining precise adherence to the desired.
Oscillation Control	Frequent and noticeable oscillations, particularly after rapid changes.	Reduces oscillations compared to PID but does not fully eliminated.	Effectively reduces oscillations, resulting in more stable response.	Nearly eliminates oscillations, achieving the most stable and controlled response.
Settling Pattern	Takes longer time to stabilize, with noticeable overshoot and oscillations.	Stabilizes faster than PID, with minor overshoot.	Settles quickly, with minimal overshoot and good alignment to the desired.	Achieves the fastest and most accurate settling, with minimal overshoot and precise tracking.

End-effector position error is a key indicator of the effectiveness of the control strategy used for the Delta robot. In this study, the position errors in the X, Y, and Z directions are used to compare the performance of HS-optimized PID, HS-optimized TID, Self-tuning Fuzzy PID, and Self-tuning Fuzzy TID controllers. This evaluation provides insight into the accuracy and overall improvement of the proposed control methods.

**Fig. 23:**
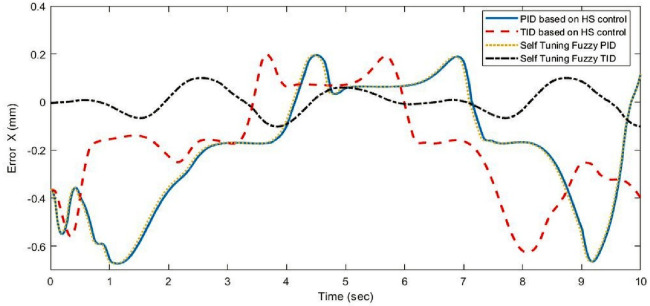
Error of end effector in x-axis of Responses for PID & TID based on HS, Fuzzy PID & Fuzzy TID for Sine input.

Figure 23 presents the X-axis position error comparison among the four controllers. The TID-based controllers, both HS-optimized and fuzzy, achieve lower error magnitudes compared to PID-based methods. In particular, the Fuzzy TID controller demonstrates nearly zero error in the X-direction, indicating the highest level of precision. These results confirm the superior accuracy of TID-based control and show that PID controllers, even with fuzzy enhancement, may require further improvement to achieve comparable performance.

**Fig. 24:**
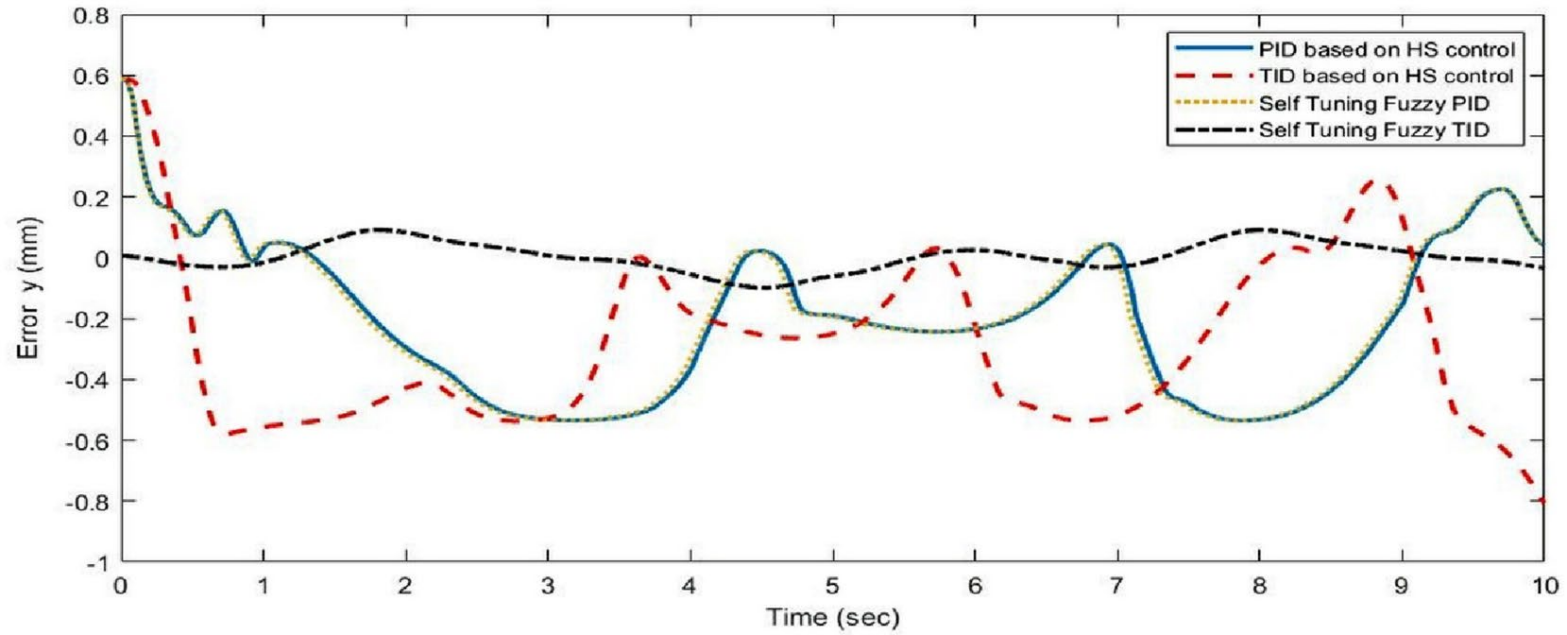
Error of end effector in y-axis of Responses for PID & TID based on HS, Fuzzy PID & Fuzzy TID for Sine input.

Figure 24 presents a comparison of the Y-axis position error (in mm) for the four controllers. The Fuzzy TID controller achieves the lowest error, which is nearly zero, followed by the HS-optimized TID controller. In contrast, the HS-optimized PID controller shows the highest error, while the Self-Tuning Fuzzy PID provides some improvement over the standard PID but still remains less accurate than the TID-based controllers. These results confirm the superior tracking accuracy of the Fuzzy TID approach in the Y-direction.

**Fig. 25:**
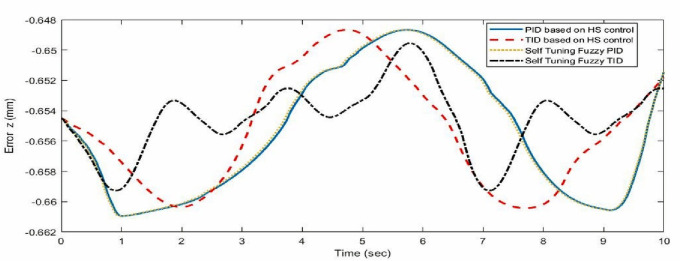
Error of end effector in z-axis of Responses for PID & TID based on HS, Fuzzy PID & Fuzzy TID for Sine input.

Figure 25 presents a comparison of the Z-axis position error (in mm) for the four controllers. The TID-based controllers, particularly the Self-Tuning Fuzzy TID, achieve lower error magnitudes and smoother tracking of the desired trajectory. In contrast, the PID-based controllers exhibit higher errors, where the Self-Tuning Fuzzy PID shows some improvement over the standard PID but still does not match the accuracy and stability of the TID controllers. These results confirm the superior performance of TID-based control in the Z-direction.

Table 10 presents a comparison of RMSE values for PID, TID, Fuzzy PID, and Fuzzy TID controllers across the X, Y, and Z axes. In the X-axis, the Fuzzy TID achieves the lowest RMSE (0.2602 mm), followed closely by TID (0.2739 mm), indicating superior precision. In the Y-axis, TID and Fuzzy TID show nearly identical performance (0.3047 mm and 0.3046 mm), both outperforming PID and Fuzzy PID. For the Z-axis, all controllers yield similar RMSE values, with Fuzzy TID achieving the smallest error (0.6548 mm). Overall, the Fuzzy TID controller provides the best or comparable accuracy across all axes, making it the most effective method for minimizing position tracking errors in dynamic robotic systems.

**Table 10:** ROOT MEAN SQUARE ERRORS FOR PID & TID based on HS, Fuzzy PID & Fuzzy TID in sine input.

	PID	TID	Fuzzy PID	Fuzzy TID
RMSE_x (mm)	0.3197	0.2739	0.3183	0.2602
RMSE_y (mm)	0.3190	0.3047	0.3126	0.3046
RMSE_z (mm)	0.6555	0.6554	0.6555	0.6548

Overall, the Fuzzy TID controller demonstrates the best performance across all axes, achieving the lowest RMSE in the X-axis and Z-axis, and matching the TID controller in the Y-axis. These results confirm its superior accuracy and adaptability compared to the other methods. The TID controller also shows strong performance, particularly in the Y-axis, while the PID and Fuzzy PID controllers exhibit higher errors and comparatively lower precision.

### Repeating-sequence stair input test

Repeating stair input is used to evaluate Delta robot controllers because it effectively tests their ability to handle sudden, repeated changes in reference signals. This input allows assessment of key performance aspects such as precision, stability, and adaptability. It is particularly useful for analyzing how well the controller minimizes overshoot, suppresses oscillations, and achieves fast stabilization after each step transition.

**Fig. 26:**
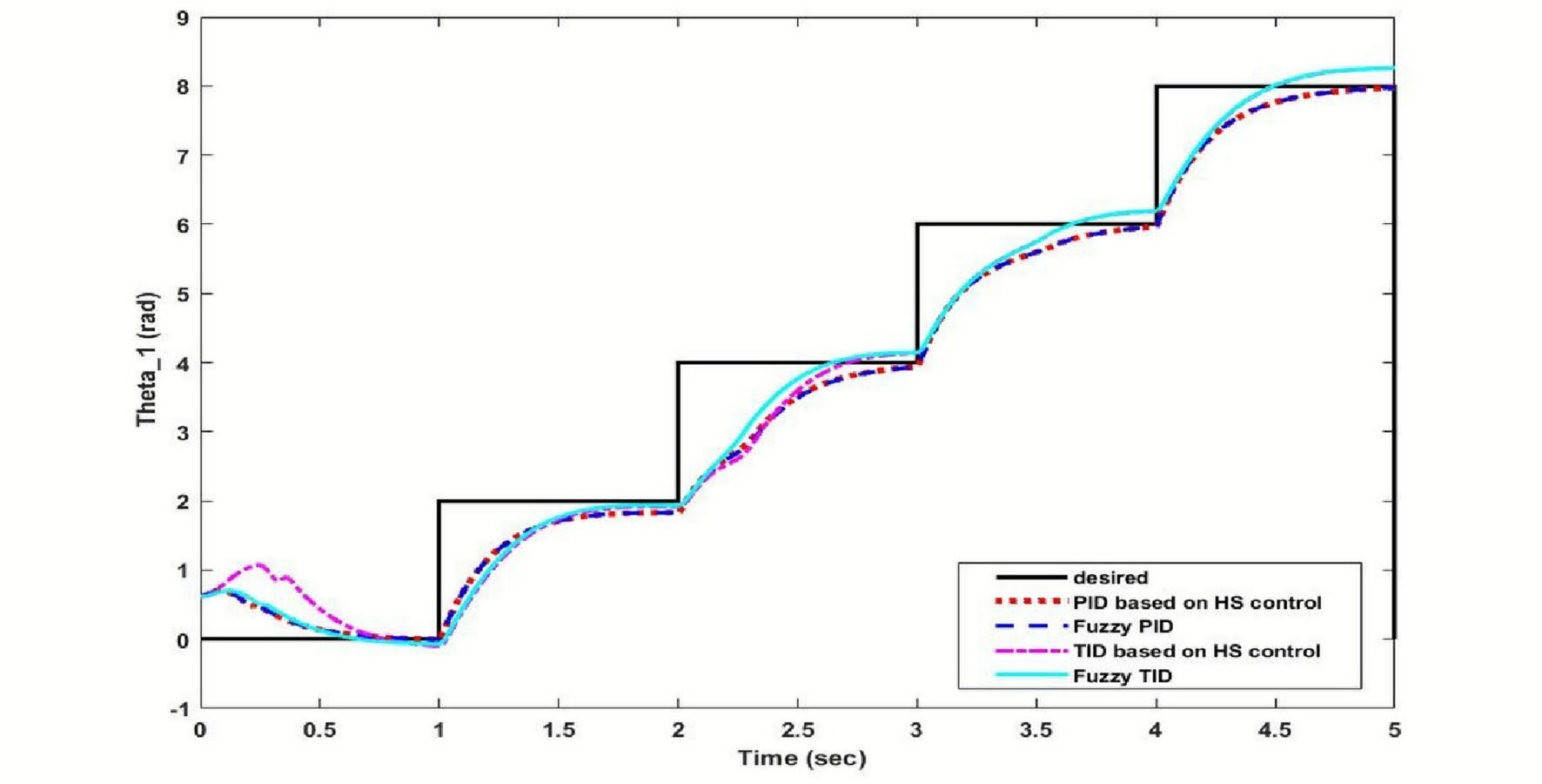
Comparison for theta_1 response between PID & TID Based on HS, Fuzzy PID &Fuzzy TID for Stair Input.

Figure 26 presents a comparison of the responses of PID, TID, Fuzzy PID, and Fuzzy TID controllers for the first joint of the Delta robot (θ₁) under a repeating stair input. All controllers are able to track the desired trajectory; however, the Fuzzy PID and Fuzzy TID controllers show smoother transitions with reduced deviations. The PID and TID controllers exhibit slight overshoot and oscillations during step changes. Among all methods, the Fuzzy TID controller achieves the best performance, providing the most accurate tracking, minimal oscillations, and faster stabilization, making it the most effective for precise and stable control.

Across all phases (0–5 s), the controllers show different tracking behaviors under stair input for θ₁. In the initial phase, PID exhibits slow response and overshoot, Fuzzy PID improves speed but still overshoots, and TID shows faster response with noticeable oscillations, while Fuzzy TID provides the smoothest and most accurate tracking. During the middle phase, PID and TID suffer from phase lag and oscillations, Fuzzy PID reduces these effects, and Fuzzy TID maintains the best stability and alignment with the reference trajectory. In the final phase, PID and TID show significant instability, Fuzzy PID performs moderately, while Fuzzy TID continues to deliver the most stable and accurate response with minimal deviations. Overall, the Fuzzy TID controller consistently outperforms the others, achieving superior stability, minimal overshoot, and the best trajectory tracking. The comparison is summarized in Table 11.

**Table 11:** Summary of comparison between PID&TID based on HS, Fuzzy PID and Fuzzy TID control of theta_1 performance for stair input.

	PID control	TID control	Fuzzy PID control	Fuzzy TID control
Response Smoothness	Shows moderate smoothness but exhibits some irregularities during transitions.	Smoother than PID, with reduced sudden changes.	Same as PID in this case.	Achieves the smoothest response, with the best alignment to the desired.
Stability	Moderate stability, struggles during rapid changes, with some lag.	Better stability than PID but still shows minor deviations.	Same as PID in this case.	Excellent stability, consistently adhering to the desired trajectory without significant deviations.
Oscillation Control	Noticeable oscillations after transitions, particularly at the start.	Reduces oscillations compared to PID but still has minor fluctuations.	Same as PID in this case.	Nearly eliminates oscillations, offering the most controlled response across transitions.
Settling Pattern	Slower settling, with overshoot and steps are not aligned consistently.	Faster settling than PID, with reduced overshoot.	Same as PID in this case.	Achieves the fastest and most accurate settling, aligning to the desired steps without overshoot.

**Fig. 27:**
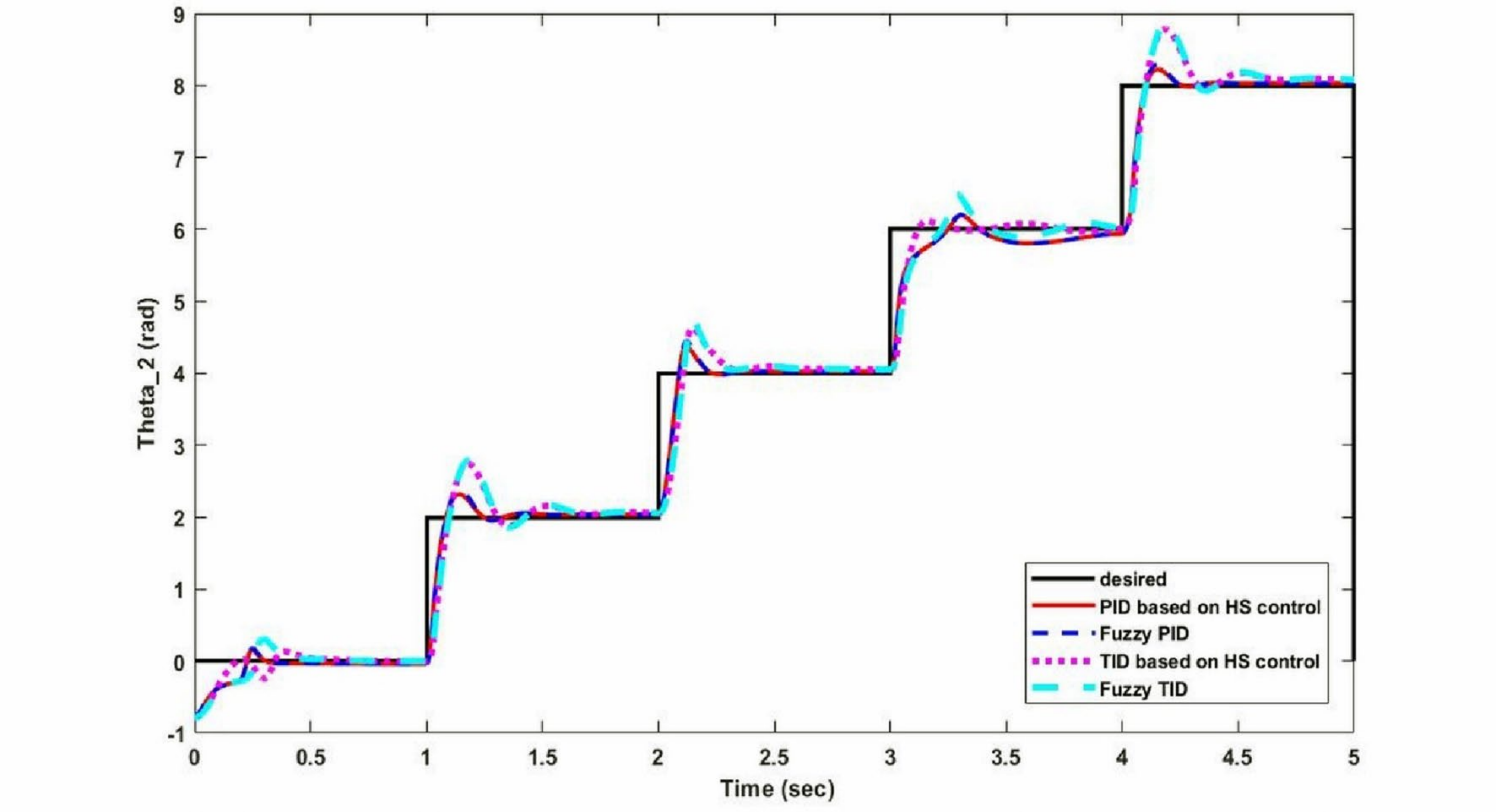
Comparison for theta_2 response between PID & TID Based on HS, Fuzzy PID &Fuzzy TID for Stair Input.

Figure 27 presents a comparison of the responses of PID, TID, Fuzzy PID, and Fuzzy TID controllers for the second joint of the Delta robot (θ₂) under stair input conditions. All controllers are able to follow the desired trajectory; however, the Fuzzy PID and Fuzzy TID controllers exhibit smoother and more accurate transitions. In contrast, the PID and TID controllers show more noticeable oscillations and overshoot during step changes, particularly in the initial and middle phases.

Across all phases (0–5 s), the controllers exhibit different tracking behaviors for θ₂ under stair input. In the initial phase, PID shows slow response with overshoot and oscillations, Fuzzy PID improves speed but still overshoots, and TID provides faster response but with instability, while Fuzzy TID achieves the best tracking with minimal overshoot and smooth transitions. During the middle phase, PID and TID show phase lag and oscillations, Fuzzy PID reduces these effects, and Fuzzy TID maintains the highest accuracy and stability. In the final phase, PID and TID degrade further with noticeable instability, Fuzzy PID performs moderately, while Fuzzy TID continues to deliver the most stable and precise response. Overall, the Fuzzy TID controller consistently outperforms the others in terms of accuracy, stability, and tracking performance. The comparison is summarized in Table 12.

**Table 12:** Summary comparison of θ₂ performance for HS-optimized PID, HS-optimized TID, Fuzzy PID, and Fuzzy TID controllers under stair input.

	PID control	TID control	Fuzzy PID control	Fuzzy TID control
Response Smoothness	Moderate smoothness but shows irregular transitions between steps.	Smoother than PID, with less abrupt variations.	Same as PID in this case.	Exhibits the highest level of smoothness, closely aligning with the desired trajectory.
Stability	Struggles with stability, especially during transitions, with noticeable oscillations.	Improved stability compared to PID, but still displays slight deviations.	Same as PID in this case.	Outstanding stability, following the desired accurately with minimal deviations.
Oscillation Control	Noticeable oscillations after step transitions, leading to delayed responses.	Reduces oscillations compared to PID but still has minor fluctuations.	Same as PID in this case.	Effectively eliminates oscillations, providing the most controlled and reliable response.
Settling Pattern	Slower settling, with some overshoot and inconsistencies aligning to the step pattern.	Settles faster than PID, with reduced overshoot.	Same as PID in this case.	Achieves the fastest and most accurate settling, aligning perfectly with the desired steps.

**Fig. 28:**
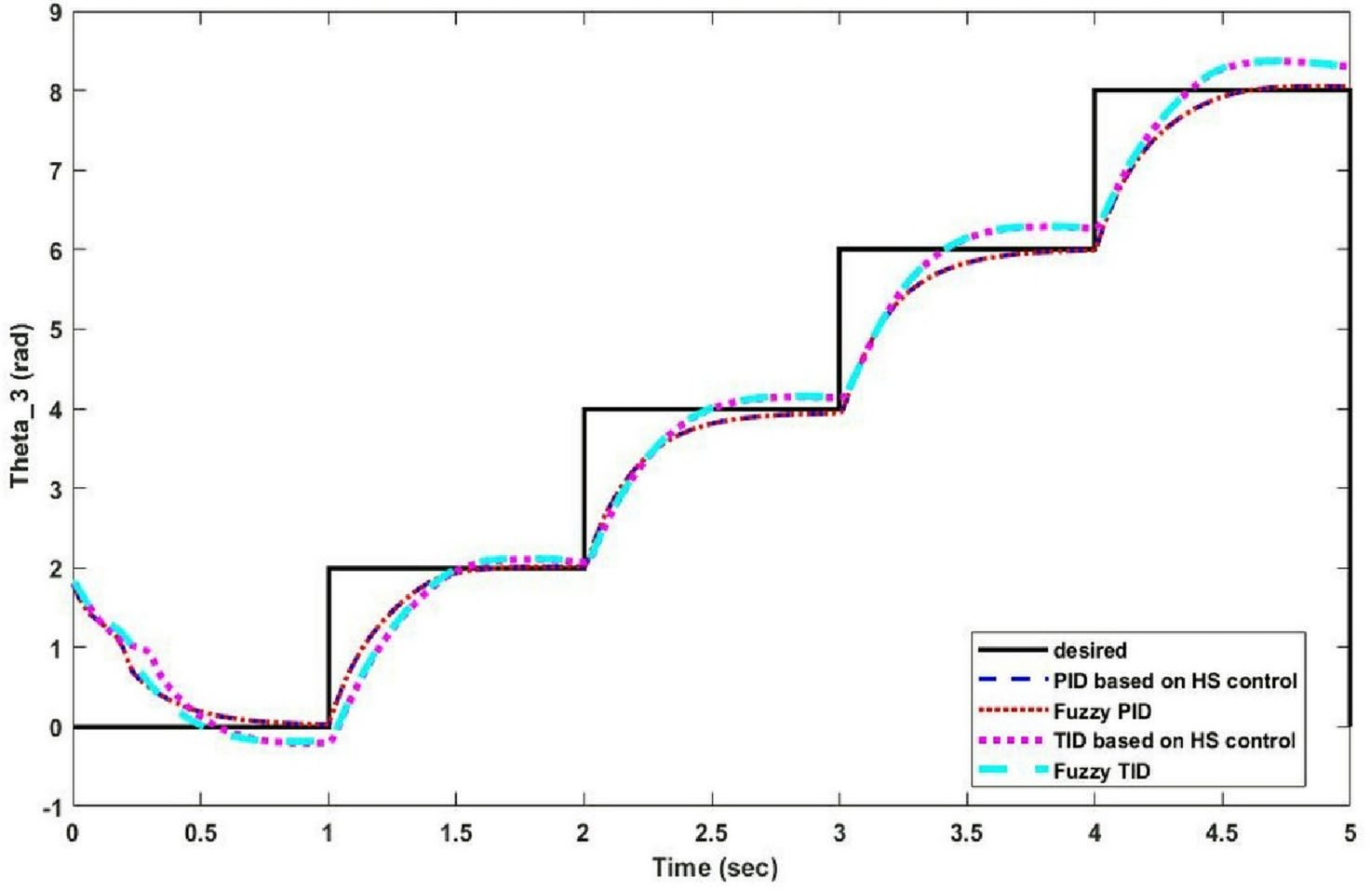
Comparison for theta_3 response between PID & TID Based on HS, Fuzzy PID &Fuzzy TID for Stair Input.

Figure 28 compares the responses of PID, TID, Fuzzy PID, and Fuzzy TID controllers for the third joint of the Delta robot (θ₃) under stair input conditions. All controllers successfully track the desired trajectory; however, Fuzzy PID and Fuzzy TID provide smoother and more accurate responses than PID and TID. The PID and TID controllers show noticeable oscillations and overshoot, especially during the initial and mid-phase transitions. Among all methods, the Fuzzy TID controller achieves the best performance, with minimal overshoot, faster stabilization, and the closest tracking of the reference trajectory, making it the most suitable for high-precision and smooth control applications.

Across all phases (0–5 s), the controllers show different tracking behaviors for θ₃ under stair input. In the initial phase, PID exhibits slow response with overshoot and oscillations, Fuzzy PID improves response speed but still overshoots, and TID responds faster but with reduced stability, while Fuzzy TID achieves the best tracking with minimal overshoot and smooth transitions. During the middle phase, PID and TID show phase lag and oscillations, Fuzzy PID reduces these effects, and Fuzzy TID maintains the highest accuracy and stability. In the final phase, PID and TID performance deteriorates further with noticeable instability, Fuzzy PID performs moderately, while Fuzzy TID continues to deliver the most stable and precise response. Overall, the Fuzzy TID controller consistently outperforms the others in terms of stability, accuracy, and tracking performance. The comparison is summarized in Table 13.

**Table 13:** Summary of comparison between PID&TID based on HS, Fuzzy PID and Fuzzy TID control of theta_3 performance for stair input.

	PID control	TID control	Fuzzy PID control	Fuzzy TID control
Response Smoothness	During step transitions, there are little errors but overall the smoothness is moderate.	Improved smoothness compared to PID, with fewer sudden variations.	Same as PID in this case.	Demonstrates the highest level of smoothness, closely following the desired with minimal deviations.
Stability	Struggles with stability during transitions, showing noticeable oscillations.	Improved stability compared to PID, but still exhibits minor deviations during transitions.	Same as PID in this case.	Outstanding stability, with precise to the desired and almost no deviations.
Oscillation Control	Noticeable oscillations, particularly after step changes, leading to slower stabilization.	Reduces oscillations compared to PID but retains some minor fluctuations.	Same as PID in this case.	Effectively eliminates oscillations, providing the most controlled and reliable response during and after transitions.
Settling Pattern	Slower settling with some overshoot and delayed alignment to the step pattern.	Faster settling than PID, with reduced overshoot and quicker stabilization.	Same as PID in this case.	Achieves the fastest and most accurate settling, perfectly following the step pattern with no noticeable deviations

The End-effector error is important for evaluating the best controller because it provides a direct measure of how accurately each controller can handle rapid changes in trajectory, which are common in real-world applications. Controllers with lower RMSE values demonstrate superior tracking accuracy and better adaptation to abrupt changes, making them more reliable for applications requiring high precision, such as robotic manipulation or industrial automation.

**Fig. 29:**
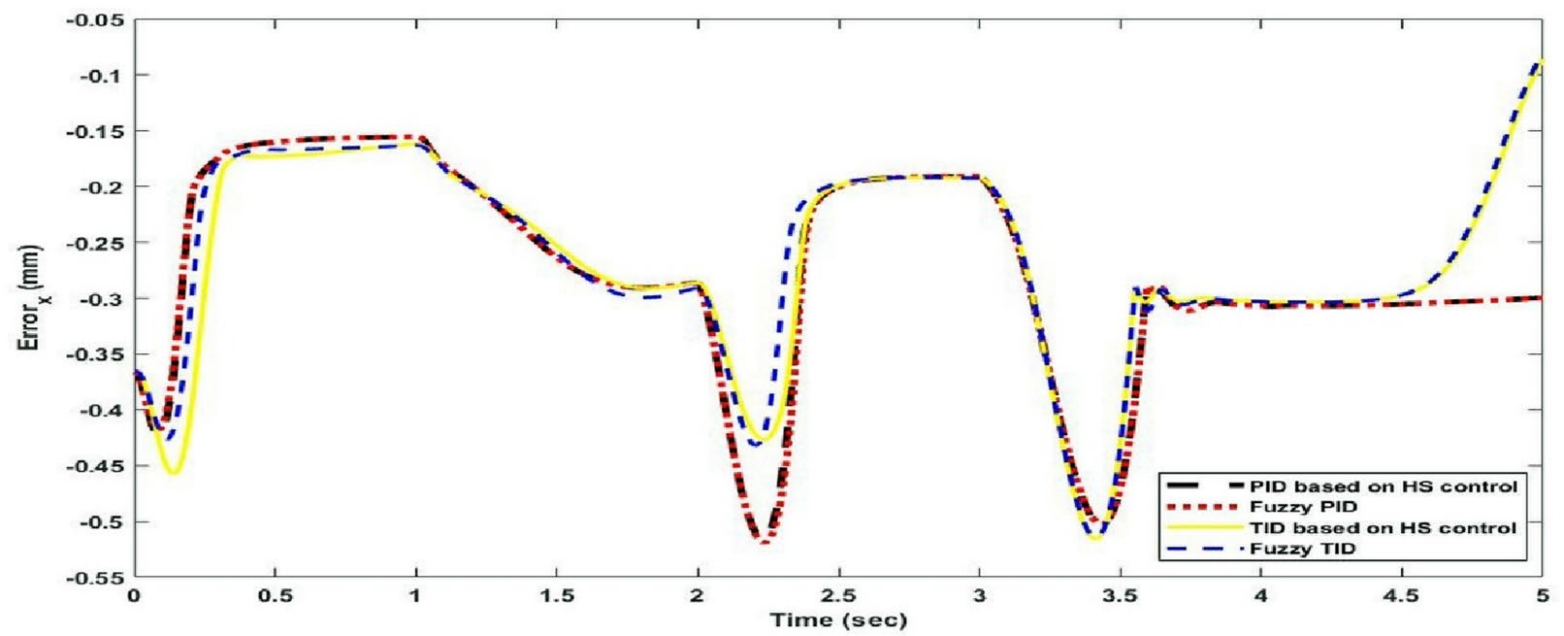
Error of end effector in x-axis of Responses for PID & TID based on HS, Fuzzy PID & Fuzzy TID for Stair input.

Figure 29 compares the error in the X-axis (in mm) for four controllers. The Fuzzy TID controller demonstrates the lowest error magnitude, with minimum error throughout the time range, indicating excellent precision. The TID based on HS control also exhibits low error magnitudes, though slightly higher than the Fuzzy TID. In contrast, the PID controllers (both traditional and fuzzy) show higher error magnitudes and are less consistent in tracking the desired trajectory.

**Fig. 30:**
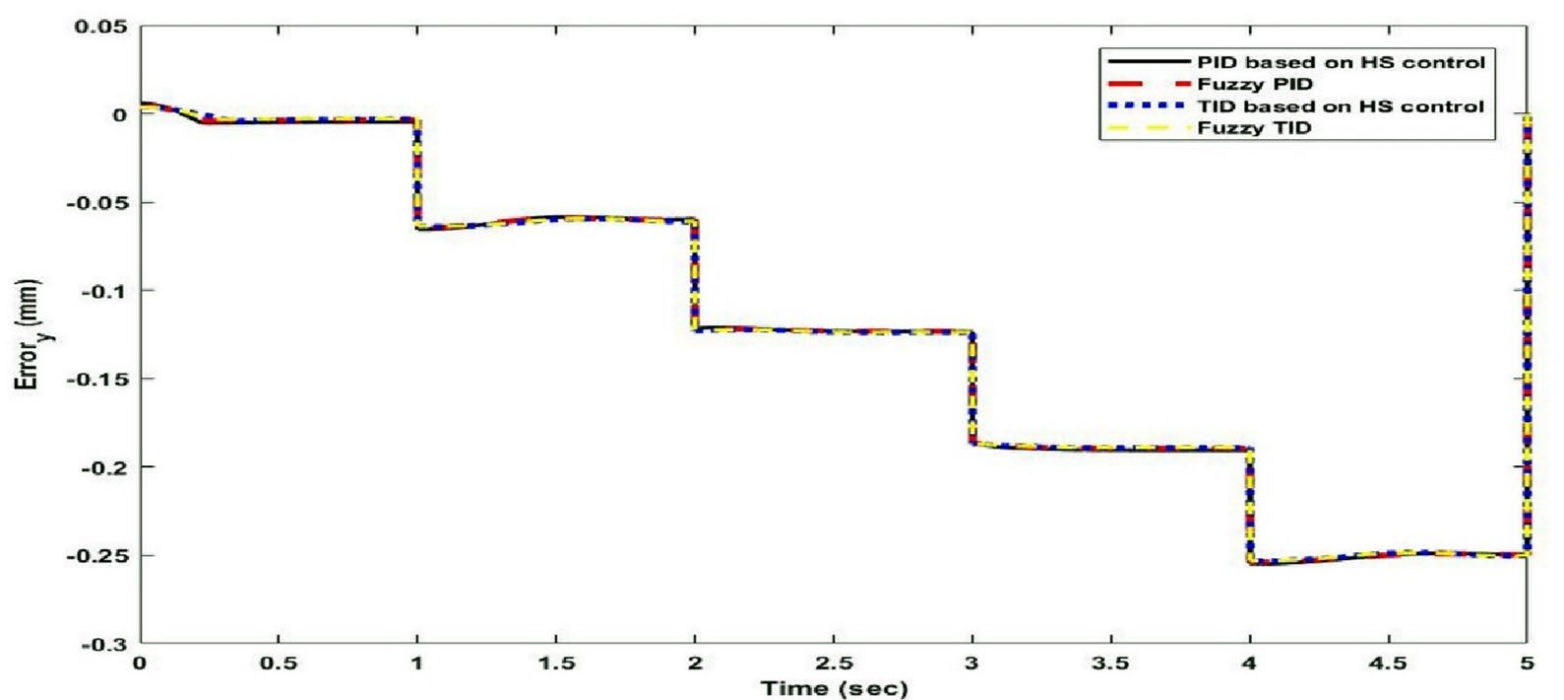
Error of end effector in y-axis of Responses for PID & TID based on HS, Fuzzy PID & Fuzzy TID for Stair input.

Figure 30 compares the error in the Y-axis (in mm) for four controllers. All controllers demonstrate nearly identical performance, with minimal error magnitudes throughout the time range. The errors are closely aligned for all four methods, indicating a high level of precision and stability across the board.

**Fig. 31:**
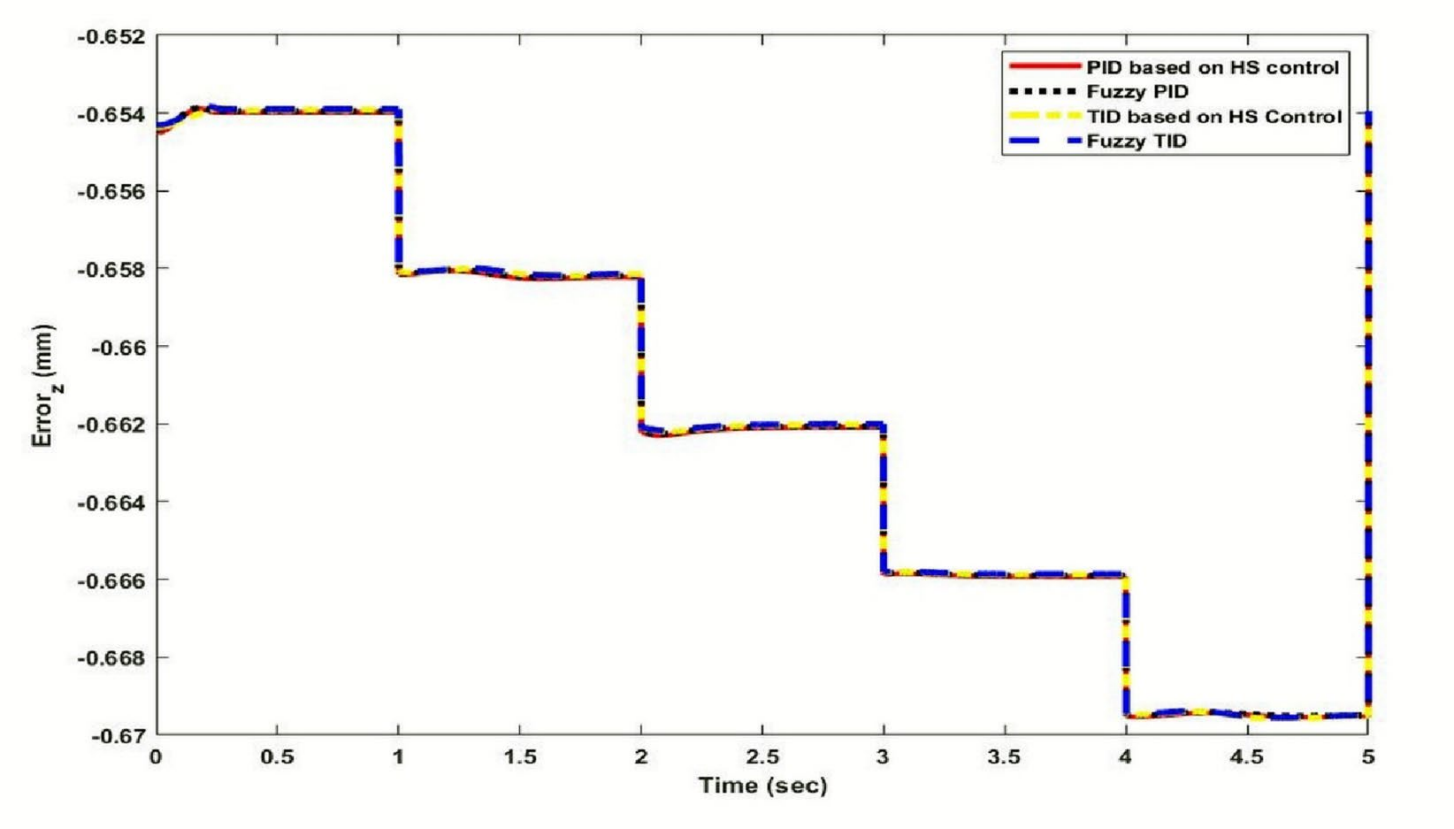
Error of end effector in z-axis of Responses for PID & TID based on HS, Fuzzy PID & Fuzzy TID for Stair input.

While Fig. 31 compares the error in the Z-axis (in mm) for four controllers. All four controllers exhibit nearly identical errors throughout the test duration. The error magnitudes are extremely close, with no significant differences between the methods.

Table 14 summarizes the RMSE values for PID, TID, Fuzzy PID, and Fuzzy TID controllers across the X, Y, and Z axes. In the X-axis, the Fuzzy TID achieves the lowest RMSE (0.2761 mm), followed by TID (0.2805 mm), while PID and Fuzzy PID show slightly higher errors. For the Y-axis, all controllers perform similarly, with Fuzzy TID providing the smallest RMSE (0.1530 mm). In the Z-axis, differences are minimal, but Fuzzy TID still attains the lowest error (0.6617 mm). Overall, the Fuzzy TID controller delivers the best accuracy across all axes, making it the most effective approach for high-precision applications. The Fuzzy TID controller achieves the lowest RMSE and best overall accuracy, outperforming the other methods, while PID-based controllers show the highest errors.

**Table 14:** ROOT MEAN SQUARE ERRORS FOR PID & TID based on HS, Fuzzy PID & Fuzzy TID in stair input.

	PID	TID	Fuzzy PID	Fuzzy TID
RMSE_x (mm)	0.2899	0.2805	0.2904	0.2761
RMSE_y (mm)	0.1535	0.1531	0.1532	0.1530
RMSE_z (mm)	0.6620	0.6619	0.6619	0.6617

## Consolidated discussion of repeated findings

Overall, the repeated findings can be summarized as follows: PID is the simplest baseline but has the highest tracking error; fuzzy PID improves damping and adaptability; optimized TID improves steady-state and Cartesian accuracy; and fuzzy TID provides the strongest overall tracking performance. However, optimized TID remains attractive when computational simplicity is required for real-time implementation.

Across the repeating-stair tests, the controllers are challenged by repeated abrupt reference changes. PID and TID show visible oscillation and delayed recovery after transitions, whereas fuzzy adaptation improves damping. Fuzzy TID achieves the lowest RMSE values in X, Y, and Z, indicating the best ability to recover after sudden command changes.

Across the sine-wave tests, the main limitation of PID-based control is phase lag and oscillation near transitions and peaks. TID improves tracking accuracy, but fuzzy TID provides the smoothest and most accurate trajectory following. The RMSE results confirm this trend: fuzzy TID achieves 0.2602 mm in X, 0.3046 mm in Y, and 0.6548 mm in Z, which is the best or comparable-best performance among the four controllers^75–77^.

Across the step-input tests, PID generally provides a faster response but suffers from larger steady-state error. TID reduces steady-state error considerably, especially for θ₁ and θ₃, but may increase overshoot and settling time. Fuzzy PID improves smoothness relative to PID, while fuzzy TID combines the steady-state accuracy of TID with the adaptive smoothing effect of fuzzy logic^78–86^.

## Conclusion

The Delta robot is widely employed in high-speed and precision applications, where accurate trajectory tracking is challenging because of nonlinear kinematics, coupled dynamics, and practical mechanical effects. This study presented a comparative evaluation of HS-optimized PID, HS-optimized TID, fuzzy PID, and fuzzy TID controllers using a validated Simscape-based Delta robot model that incorporates nonlinear effects, including friction and backlash. The controllers were evaluated under step, sinusoidal, and repeating-stair reference trajectories to assess their tracking performance.

The simulation results indicate that the conventional PID controller provides a simple baseline solution, while TID-based controllers generally improve tracking accuracy and reduce steady-state error under several operating conditions. Among the evaluated methods, the fuzzy TID controller achieved the lowest or near-lowest Cartesian RMSE for the considered dynamic trajectories, demonstrating the best overall tracking performance within the scope of this study. These improvements are attributed to the combined effect of Harmony Search-based parameter optimization and fuzzy adaptation, which enhance the controller’s ability to accommodate the nonlinear characteristics of the Delta robot model.

The findings suggest that optimized PID/TID-based controllers can provide accurate trajectory tracking with relatively low online computational complexity for the investigated simulation scenarios. However, the conclusions are limited to the validated Simscape model and the reference trajectories considered in this work. Although the model incorporates practical nonlinearities such as friction and backlash, external disturbances, measurement noise, payload variations, and hardware implementation were not investigated. Consequently, the reported performance should be interpreted within the scope of the simulation-based evaluation.

Future work will focus on experimental validation using a physical Delta robot platform, as well as robustness assessment under external disturbances, parameter uncertainties, varying payloads, and sensor noise to further evaluate the practical applicability of the proposed control strategies.

## Data Availability

No datasets were generated or analysed during the current study.

## Abbreviations

PID: Proportional-Integral-Derivative controller
TID: Tilt-Integral-Derivative controller
HSO/HS: Harmony Search Optimization / Harmony Search
NLARX: Nonlinear autoregressive model with exogenous inputs
RMSE: Root mean square error
MSE: Mean square error
MIMO: Multi-input multi-output system
HM, HMS, HMCR, PAR, BW: Harmony memory, harmony memory solution, harmony memory consideration rate, pitch adjustment rate, and bandwidth factor

## Code availability

The MATLAB/Simulink models and scripts used in this study are available from the corresponding author upon reasonable request.

## Declarations

### Objective and scope

This study aims to develop and compare four control strategies for a Delta robot: HS-optimized PID, HS-optimized TID, self-tuning fuzzy PID, and self-tuning fuzzy TID. The comparison covers model development, nonlinear system identification, offline Harmony Search tuning, and joint-space and Cartesian tracking under step, sinusoidal, and repeating-stair references.
